# Minocycline counter-regulates pro-inflammatory microglia responses in the retina and protects from degeneration

**DOI:** 10.1186/s12974-015-0431-4

**Published:** 2015-11-17

**Authors:** Rebecca Scholz, Markus Sobotka, Albert Caramoy, Thomas Stempfl, Christoph Moehle, Thomas Langmann

**Affiliations:** Laboratory for Experimental Immunology of the Eye, Department of Ophthalmology, University of Cologne, 50931 Cologne, Germany; Center of Excellence for Fluorescent Bioanalytics, University of Regensburg, 93053 Regensburg, Germany

**Keywords:** Minocycline, Microglia, Photoreceptors, Retinal degeneration, Light damage, Age-related macular degeneration

## Abstract

**Background:**

Microglia reactivity is a hallmark of retinal degenerations and overwhelming microglial responses contribute to photoreceptor death. Minocycline, a semi-synthetic tetracycline analog, has potent anti-inflammatory and neuroprotective effects. Here, we investigated how minocycline affects microglia in vitro and studied its immuno-modulatory properties in a mouse model of acute retinal degeneration using bright white light exposure.

**Methods:**

LPS-treated BV-2 microglia were stimulated with 50 μg/ml minocycline for 6 or 24 h, respectively. Pro-inflammatory gene transcription was determined by real-time RT-PCR and nitric oxide (NO) secretion was assessed using the Griess reagent. Caspase 3/7 levels were determined in 661W photoreceptors cultured with microglia-conditioned medium in the absence or presence of minocycline supplementation. BALB/cJ mice received daily intraperitoneal injections of 45 mg/kg minocycline, starting 1 day before exposure to 15.000 lux white light for 1 hour. The effect of minocycline treatment on microglial reactivity was analyzed by immunohistochemical stainings of retinal sections and flat-mounts, and messenger RNA (mRNA) expression of microglia markers was determined using real-time RT-PCR and RNA-sequencing. Optical coherence tomography (OCT) and terminal deoxynucleotidyl transferase dUTP nick end labeling (TUNEL) stainings were used to measure the extent of retinal degeneration and photoreceptor apoptosis.

**Results:**

Stimulation of LPS-activated BV-2 microglia with minocycline significantly diminished the transcription of the pro-inflammatory markers CCL2, IL6, and inducible nitric oxide synthase (iNOS). Minocycline also reduced the production of NO and dampened microglial neurotoxicity on 661W photoreceptors. Furthermore, minocycline had direct protective effects on 661W photoreceptors by decreasing caspase 3/7 activity. In mice challenged with white light, injections of minocycline strongly decreased the number of amoeboid alerted microglia in the outer retina and down-regulated the expression of the microglial activation marker translocator protein (18 kDa) (TSPO), CD68, and activated microglia/macrophage whey acidic protein (AMWAP) already 1 day after light exposure. Furthermore, RNA-seq analyses revealed the potential of minocycline to globally counter-regulate pro-inflammatory gene transcription in the light-damaged retina. The severe thinning of the outer retina and the strong induction of photoreceptor apoptosis induced by light challenge were nearly completely prevented by minocycline treatment as indicated by a preserved retinal structure and a low number of apoptotic cells.

**Conclusions:**

Minocycline potently counter-regulates microgliosis and light-induced retinal damage, indicating a promising concept for the treatment of retinal pathologies.

**Electronic supplementary material:**

The online version of this article (doi:10.1186/s12974-015-0431-4) contains supplementary material, which is available to authorized users.

## Background

Age-related macular degeneration (AMD) is a leading cause of severe visual impairment in the elderly, and the number of affected persons steadily increases as a consequence of demographic changes [1]. Late stage AMD can be classified in a dry and a wet form, representing atrophic and neovascular processes, respectively. Wet AMD can be treated with intravitreal injections of anti-vascular endothelial growth factor (VEGF) medication, while no established treatment options exist for the dry form [2]. The pathogenesis of AMD is characterized by the early presence of drusen, damage of the retinal pigment epithelium (RPE) and photoreceptor layer primarily affecting the macular region and a chronic inflammatory response in the retina leading to the expression of pro-inflammatory and pro-angiogenic factors [2]. This immunological response in the retina can be regarded as para-inflammation and involves the reactivity of microglial cells [3–5].

Microglial cells are the tissue macrophages of the central nervous system (CNS), including the retina. In the healthy adult retina, they are located in the plexiform layers from where they permanently scan the retinal environment with their motile protrusions [6]. The expression of several receptors that are specific for the binding of chemokines, cytokines, complement factors, antibodies, or damage-associated molecular patterns enables these cells to recognize and immediately respond to pathological changes of their environment [7–9]. Besides their supportive function in the healthy retina, microglia reactivity and age-related changes of microglia physiology contribute to degenerative pathologies of the retina and the entire CNS [3, 4, 8, 10–17]. A large number of amoeboid shaped reactive microglia are detectable in the degenerating photoreceptor layer of AMD and retinitis pigmentosa retinas [4]. These cells contain phagocytosed rhodopsin-positive particles [4], and a recent report demonstrated that microglia do not only phagocytose dead cells but also take up living rods in a mouse model for retinitis pigmentosa [13]. Therefore, microglial activation cannot be just regarded as a bystander effect but rather actively contributes to photoreceptor cell death during retinal degeneration.

Because of these findings, substances that modulate microglial reactivity such as minocycline are good candidates to prevent inflammation and dampen degenerative processes in the retina. Minocycline is a second-generation semi-synthetic tetracycline analog, which is used against Gram-positive and Gram-negative bacteria for over 30 years. Besides its bacteriostatic capacity, minocycline exerts anti-inflammatory, anti-apoptotic, and neuroprotective effects in experimental models of Parkinson’s disease [18], multiple sclerosis [19–22], neuropathic pain [16], Alzheimer’s disease [23, 24], and in a genetic mouse model of retinitis pigmentosa [25].

In this study, we addressed the questions how minocycline modulates microglial reactivity in vitro and whether it protects from acute white light-induced retinal degeneration in the mouse. We selected white light exposure as it is an environmental risk factor that mimics several features of AMD in rodents including degeneration of photoreceptors and the retinal pigment epithelium [26–28]. This model is also very useful for a temporal correlation of microglial responses with processes of retinal degeneration and RPE dysfunction [29–31].

## Methods

### Reagents

Minocycline hydrochloride (M9511), *Escherichia coli* 0111:B4 lipopolysaccharide (LPS), and Z-Leu-Leu-Leu-al (MG-132) were purchased from Sigma-Aldrich (St. Louis, MO, USA).

### Cell culture

BV-2 microglia were cultured in RPMI1640 with 5 % fetal calf serum (FCS) supplemented with 2 mM l-glutamine, 1 % penicillin/streptomycin, and 195 nM β-mercaptoethanol at 37 °C in a humidified atmosphere of 5 % CO_2_, as described previously [32, 33]. BV-2 cells were pre-incubated for 30 min with 50 μg/ml minocycline or NaCl as vehicle control. Afterwards, the cells were stimulated with 50 ng/ml LPS, 50 μg/ml minocycline, or 50 ng/ml LPS plus 50 μg/ml minocycline for 24 h. After stimulation, cells or supernatant were harvested for further analysis. 661W photoreceptor-like cells were a kind gift from Prof. Muayyad Al-Ubaidi (Department of Cell Biology, University of Oklahoma Health Sciences Center, Oklahoma City, OK, USA). 661W photoreceptor cells were cultured with Dulbecco’s modified Eagle’s medium (DMEM), high glucose with l-glutamine, supplemented with 10 % FCS and 1 % penicillin/streptomycin. Cultures were maintained in a sterile humidified environment at 37 °C and 5 % CO_2_ as described elsewhere [34].

### RNA isolation and reverse transcription

Total RNA was extracted from cultured BV-2 microglia or murine retinas using the NucleoSpin® RNA Mini Kit (Macherey-Nagel, Dueren, Germany). RNA was quantified spectrophotometrically with a NanoDrop 2000 (Thermo Scientific). First-strand complementary DNA (cDNA) synthesis was carried out with the Revert Aid H Minus First-strand cDNA Synthesis Kit (Fermentas, K1632).

### Real-time RT-PCR

cDNA (25 ng) were amplified in a 10-μl reaction mixture consisting of 5 μl Fast Start Universal Probe Master (Rox) (Roche), 2 μl of primers (10 μM), 0.375 μl purified water, and 0.125 μl of dual-labeled Universal ProbeLibrary (UPL) probe (Roche Applied Science, Basel, Switzerland) with an Applied Biosystems 7900 HT Fast Real-time PCR system (Applied Biosystems, Carlsbad, CA, USA). The following reaction parameters were used: 10 min 95 °C hold, followed by 40 cycles of 15 s 95 °C melt, and 1 min 60 °C anneal/extension. Primer sequences and UPL probe numbers were as follows: CD68, forward primer 5′-ctctctaaggctacaggctgct-3′, reverse primer 5′-tcacggttgcaagagaaaca-3′, probe #27; activated microglia/macrophage whey acidic protein (AMWAP), forward primer 5′-tttgatcactgtggggatga-3′, reverse primer 5′-acactttctggtgaaggcttg-3′, probe #1; translocator protein (TSPO), forward primer 5′-actgtattcagccatggggta-3′, reverse primer 5′-accatagcgtcctctgtgaaa-3′, probe #33; IL6, forward primer 5′-gatggatgctaccaaactggat-3′, reverse primer 5′-ccaggtagctatggtactccaga-3′, probe #6; inducible nitric oxide synthase (iNOS), forward primer 5′-ctttgccacggacgagac-3′, reverse primer 5′-tcattgtactctgagggctga-3′, probe #13; CCL2, forward primer 5′-catccacgtgttggctca-3′, reverse primer 5′-gatcatcttgctggtgaatgagt-3′, probe #62; CASP8, forward primer 5′-tgaacaatgagatccccaaat-3′, reverse primer 5′-caaaaatttcaagcaggctca-3′, probe #11; and ATP5B, forward primer 5′-ggcacaatgcaggaaagg-3′, reverse primer 5′-tcagcaggcacatagatagcc-3′, probe #77. Measurements were performed in triplicates. ATP5B expression was used as reference gene and results were analyzed with the ABI sequence detector software version 2.4 using the ΔΔCt method for relative quantification.

### RNA-sequencing and bioinformatic data analysis

Library preparation and RNA-seq were carried out as described in the Illumina TruSeq Stranded mRNA Sample Preparation Guide, the Illumina HiSeq 1000 System User Guide (Illumina, Inc., San Diego, CA, USA), and the KAPA Library Quantification Kit—Illumina/ABI Prism User Guide (Kapa Biosystems, Inc., Woburn, MA, USA). In brief, 300 ng of total RNA was used for purifying the poly-A containing messenger RNA (mRNA) molecules using poly-T oligo-attached magnetic beads. Following purification, the mRNA was fragmented to an average insert size of 200–400 bases using divalent cations under elevated temperature (94 °C for 4 min). The cleaved RNA fragments were copied into first-strand cDNA using reverse transcriptase and random primers. Strand specificity was achieved by replacing dTTP with dUTP in the Second Strand Marking Mix (SMM), followed by second-strand cDNA synthesis using DNA Polymerase I and RNase H. The incorporation of dUTP in second-strand synthesis quenches the second strand during amplification, because the polymerase used in the assay is not incorporated past this nucleotide. The addition of Actinomycin D to First Stand Synthesis Act D mix (FSA) prevents spurious DNA-dependent synthesis, while allowing RNA-dependent synthesis, improving strand specificity. These cDNA fragments then had the addition of a single “A” base and subsequent ligation of the adapter. The products were purified and enriched with PCR to create the final cDNA library. The libraries were quantified using the KAPA SYBR FAST ABI Prism Library Quantification Kit. Equimolar amounts of each library were used for cluster generation on the cBot (TruSeq SR Cluster Kit v3). The sequencing run was performed on a HiSeq 1000 instrument using the indexed, 50 cycles single read (SR) protocol and the TruSeq SBS v3 Kit. Image analysis and base calling resulted in .bcl files, which were converted into .fastq files by the CASAVA1.8.2 software. Library preparation and RNA-seq were performed at the service facility “KFB—Center of Excellence for Fluorescent Bioanalytics” (Regensburg, Germany).

The RNA express workflow on Illumina BaseSpace was used to determine differential gene expression in two biological replicates of control retinas, light-exposed retinas, and light-exposed retinas treated with minocycline, respectively. In brief, alignment of RNA-seq reads to the mouse UCSC mm10 genome and mapping to genes was performed with STAR aligner [35]. Differential gene expression between the two biological replicates each was calculated with DESeq2 [36]. The cutoff of genes considered to be differentially expressed was a log2 fold change of ≥2 or ≤−2.

Integrative analysis of genome-wide expression activities was performed with the Gene Expression Dynamics Inspector (GEDI), a Matlab (Mathworks, Natick, MA) freeware program which uses self-organizing maps (SOMs) to translate high-dimensional data into a 2D mosaic [37]. Each tile of the mosaic represents an individual SOM cluster and is color-coded to represent high or low expression of the cluster’s genes, thus identifying the underlying pattern.

The RNA-seq raw data and normalized DESeq2 counts of this study are publicly available at the National Center for Biotechnology Information Gene Expression Omnibus (http://www.ncbi.nlm.nih.gov/geo/) as series record GSE71025.

### Nitrite measurement

Nitric oxide concentrations were determined by measurement of nitrite released into BV-2 culture supernatants using the Griess reagent system (Promega). Fifty-microliter cell culture supernatants from differentially stimulated BV-2 cells were incubated with 100 μl Griess reagent in each well of a translucent 96-well plate. After incubation for 30 min at room temperature, absorbance was measured at 540 nm on an Infinite F200 Pro plate reader (Tecan). Nitrite concentrations were calculated on the basis of a sodium nitrite reference curve.

### 661 W photoreceptor apoptosis assay

To investigate microglial neurotoxicity and to assess whether minocycline also has direct effect on photoreceptors, 661W cells were incubated for 48 h with culture supernatants from differentially stimulated BV-2 cells or culture supernatants supplemented with minocycline. Apoptotic cell death was determined using the Caspase-Glo® 3/7 Assay (Promega). Cells were lysed and incubated with a luminogenic caspase-3/7 substrate, which contains the tetrapeptide sequence DEVD. After incubation at room temperature for 1 h, the generated luminescence was measured on an Infinite F200 Pro plate reader (Tecan). A blank reaction without cells was used to determine background luminescence associated with the cell culture system and Caspase-Glo® 3/7 reagent. The values of the blank reactions were subtracted from all experimental values. Negative control reactions were performed to determine the basal caspase activity of 661 W cells. Relative luciferase units (RLU) reflect the level of apoptotic cell death.

### Animals

All experiments were performed with 10–14-weeks-old albino BALB/cJ mice of both sexes. Animals were housed in an air-conditioned environment with 12-h light-dark schedule and had free access to water and food. All experimental procedures complied with the German law on animal protection and the ARVO Statement for the Use of Animals in Ophthalmic and Vision Research. The animal protocols used in this study were reviewed and approved by the governmental body responsible for animal welfare in the state of Nordrhein-Westfalen (Landesamt für Natur, Umwelt und Verbraucherschutz Nordrhein-Westfalen, Germany) (reference number 84-02.04.2015-A039).

### Minocycline administration

The mice received intraperitoneal injections of minocycline at a dose of 45 mg/kg or NaCl as solvent control where indicated twice daily for the first two days, starting one day before the light exposure and once daily for the remaining days.

### Light exposure regimen

BALB/cJ mice were dark-adapted for 16 h before light exposure. After pupil dilatation with 1 % Phenylephrin and 2.5 % Tropicamid under dim red light, the mice were exposed to bright white light with an intensity of 15.000 lx for 1 h. After light exposure, the animals were housed in dark-reared conditions overnight and then maintained under normal light conditions for the remaining experimental period.

### Immunohistochemistry

Eyes were harvested for immunohistochemical analysis 4 days after light exposure. After fixation with 4 % paraformaldehyde, eyes were embedded in optimal cutting temperature (OCT) compound or dissected for retinal flat mount analysis. Sixteen-micrometer sections were rehydrated with PBS and blocked with dried milk solution. Flat mounts were incubated with 5 % Tween, 5 % Triton-X100 in PBS overnight and non-specific binding was blocked by incubation with dried milk solution. Subsequently, retinal sections and flat mounts were incubated with primary antibodies at 4 °C overnight. Primary antibodies targeting the following molecules were used: rabbit anti-Iba1 antibody (Wako Chemicals, Neuss, Germany), rabbit anti-TSPO antibody (Abcam, Cambridge, UK), and rabbit anti-Glial Fibrillary Acidic Protein (G9269, Sigma). After a washing step, the sections and flat mounts were incubated with a secondary antibody either conjugated to Alexa488 (green) or Alexa594 (red) (Jackson Immuno-Research, West Grove, PA, USA) for 1 h. After counterstaining with 4',6-diamidino-2-phenylindole (DAPI), the samples were mounted in DAKO fluorescent mounting medium (Dako Deutschland GmbH, Hamburg, Germany) and analyzed with an Axioskop2 MOT Plus Apotome microscope (Carl Zeiss).

### Optical coherence tomography (OCT)

Animals were anesthetized by intraperitoneal injection of Rompun (10 mg/kg body weight)-Ketavet (100 mg/kg body weight), and their pupils were dilated with Phenylephrine HCl (0.25 %)-Tropicamide (0.05 %) before image acquisition. Spectral-domain OCT (SD-OCT) was performed on both eyes with a Spectralis™ HRA + OCT device (Heidelberg Engineering) to investigate structural changes in the retina after light exposure and minocycline administration. Thickness measurements were performed with a circular ring scan (circle diameter 1, 3, 6 ETDRS), centered on the optic nerve head, which represents the average retinal thickness [μm] in a certain field. Central retinal thickness was calculated from four fields around the optic nerve head using the Heidelberg Eye Explorer Software.

### Terminal deoxynucleotidyl transferase dUTP nick end labeling (TUNEL) assay

Retinal sections were labeled with an in situ cell death detection kit, Fluorescein (Roche) to detect the amount of apoptotic cells 4 days after light exposure. For a better overview, the sections were also counterstained with DAPI for 10 min. After a washing procedure, sections were mounted in DAKO fluorescent mounting medium (Dako Deutschland GmbH, Hamburg, Germany) and analyzed with an Axioskop2 MOT Plus Apotome microscope (Carl Zeiss).

### Statistical analysis

Nitrite secretion, caspase 3/7 activities, and real-time RT-PCR data from BV-2 cells were analyzed with one-way ANOVA and Bonferroni’s multiple comparison tests. Real-time RT-PCR data of murine retinas and OCT measurements of retinal thickness were analyzed using a one-way ANOVA and Dunnett’s multiple comparison tests. *p* < 0.05 was considered statistically significant.

## Results

### Minocycline dampens the pro-inflammatory response and neurotoxic potential of microglia and promotes photoreceptor survival in vitro

**Fig. 1:**
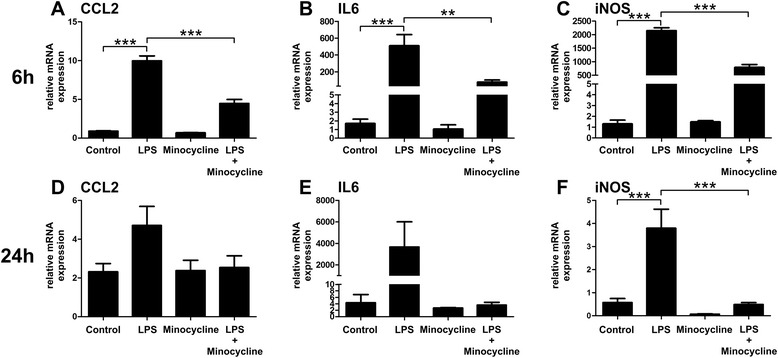
Minocycline dampens pro-inflammatory gene transcription in microglia. BV-2 microglial cells were pre-treated with 50 μg/ml minocycline or NaCl as a vehicle control for 30 min. The cells were then further stimulated with either 50 μg/ml minocycline, 50 ng/ml LPS, or a combination of both. Six hours (**a**–**c**) and 24 h (**d**–**f**) later transcript levels of the pro-inflammatory marker genes CCL2 (**a**, **d**), IL6 (**b**, **e**), and iNOS (**c**, **f**) were determined by quantitative real-time PCR. Data show mean ± SEM out of three independent experiments (*n* = 5/group, measured in triplicates) with *p < 0.05, **p < 0.01, ***p < 0.001

We first investigated whether minocycline treatment affects reactivity and neurotoxicity of BV-2 microglial cells. Gene expression of the chemoattractant protein CCL2, the pro-inflammatory cytokine IL6, and iNOS, which catalyzes the generation of toxic nitric oxide (NO), were analyzed in LPS-activated BV-2 cells that were treated with minocycline for 6 and 24 h, respectively. Minocycline significantly dampened LPS-induced gene expression of CCL2 (Fig. 1a, *p* < 0.0001 for LPS versus minocycline + LPS treatment), IL6 (Fig. 1b, *p* = 0.002 for LPS versus minocycline + LPS treatment), and iNOS (Fig. 1c, *p* < 0.0001 for LPS versus minocycline + LPS treatment) in a short-term setting of 6-h incubation. Minocycline also decreased mRNA expression of these three genes after a 24-h treatment period (Fig. 1d–f), but the level of significance was only reached for iNOS (Fig. 1f, *p* < 0.0001 for LPS versus minocycline + LPS treatment). These findings indicate that minocycline action on microglia is a relatively rapid process and very likely interferes with immediate/early mechanisms of pro-inflammatory activation.

**Fig. 2:**
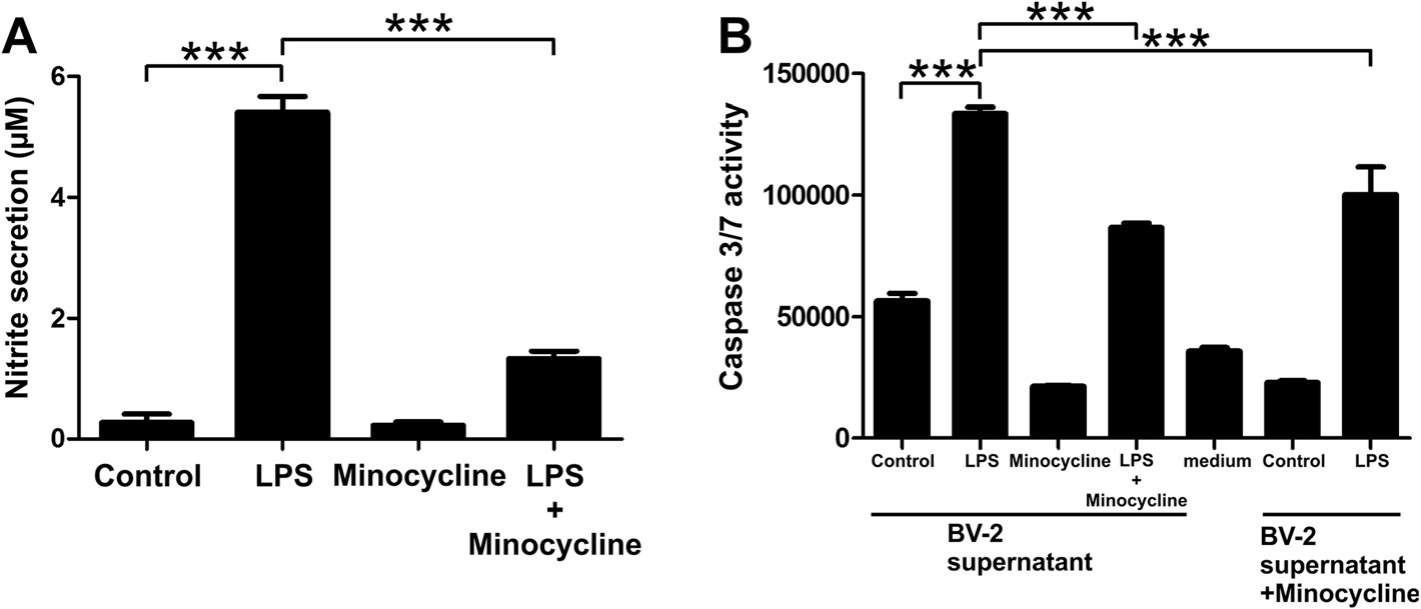
Minocycline stimulation dampens microglial NO production and reduces their neurotoxic potential on 661W photoreceptors. BV-2 cells were pre-treated with 50 μg/ml minocycline or NaCl for 30 min. The cells were then further stimulated with either 50 μg/ml minocycline, 50 ng/ml LPS, or a combination of both. **a** Twenty-four hours later, the nitric oxide production was determined by Griess reaction which detects the amount of nitrite in the cell culture supernatant. Data show mean ± SEM (*n* = 3/group, measured in triplicates). **b** To determine the neurotoxic potential of microglia, the cell culture supernatant of the differentially treated microglial cells was transferred to 661W photoreceptor cells. Furthermore, cell culture supernatant of untreated and LPS-stimulated BV-2 cells supplemented with minocycline was transferred to 661W photoreceptor cells to test whether minocycline may have a direct neuroprotective effect. After 48 h of incubation with microglia conditioned-medium, apoptotic cell death was analyzed by caspase 3/7 activity measurements. Data show mean ± SEM (*n* = 3/group, measured in duplicates) with *p < 0.05, **p < 0.01, ***p < 0.001

Since iNOS levels were also significantly inhibited by minocycline in the longer incubation experiment, we determined the amount of nitric oxide produced by BV-2 cells after stimulation with LPS, minocycline, and both compounds together after 24 h. LPS-treated microglial cells stimulated with minocycline released significantly less nitric oxide compared to cells that were stimulated with LPS alone (Fig. 2a, *p* < 0.0001), indicating that minocycline may reduce microglial neurotoxicity.

Using an apoptosis assay of 661W photoreceptor-like cells treated with microglia-conditioned medium with or without additional minocycline supplementation, we tested whether minocycline can reduce microglial neurotoxicity and possibly exert direct beneficial effects on photoreceptor survival. The supernatant from LPS-stimulated microglia significantly increased caspase 3/7 activity in 661 W cells as a biomarker of cell death (Fig. 2b, *p* < 0.0001 for LPS versus control treatment). In contrast, conditioned medium from LPS-treated BV-2 cells that were co-stimulated with minocycline induced significantly lower caspase 3/7 levels in 661 W cells (Fig. 2b, left part, *p* < 0.0001 for LPS versus minocycline + LPS supernatant treatment). Furthermore, treatment of 661W photoreceptors with supernatant from LPS-treated BV-2 cells that was supplemented with minocycline also reduced caspase 3/7 levels (Fig. 2b, right part, *p* < 0.001 for LPS versus LPS supernatant + minocycline supplement). Thus, minocycline reduced the levels of secreted neurotoxic and pro-apoptotic molecules from microglia but also exerted direct survival-promoting effects on photoreceptors that were challenged with reactive microglia-supernatants.

### Minocycline prevents microglia reactivity in retinas exposed to acute white light

**Fig. 3:**
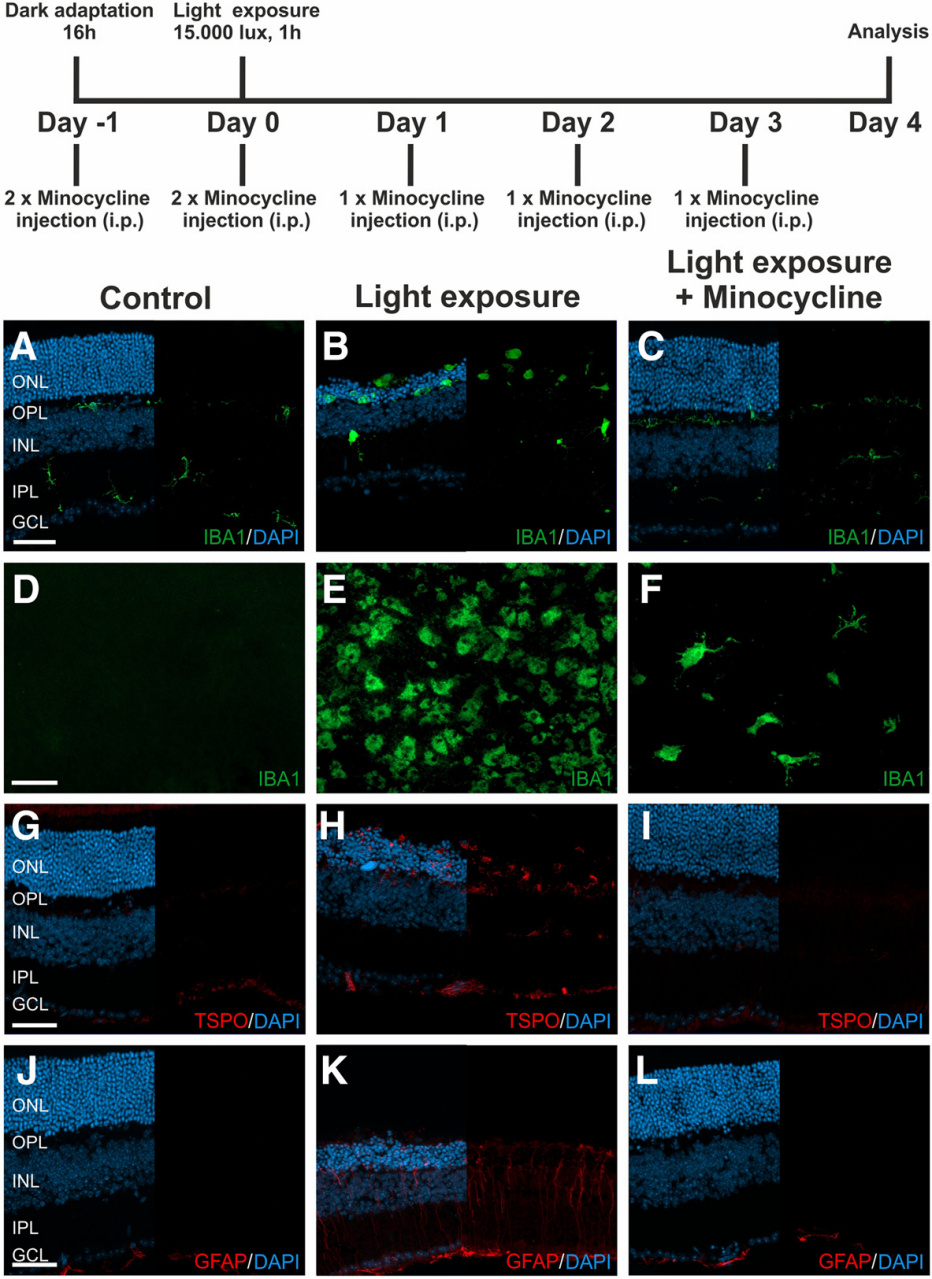
Treatment of light-exposed mice with minocycline blocks microglia reactivity and Müller cell gliosis. Of both sexes, 10- to 14-week-old BALB/c mice were dark-adapted for 16 h. After pupil dilatation, the mice were exposed to bright white light with an intensity of 15.000 lux for 1 hour. The mice received intraperitoneal injections of minocycline at a dose of 45 mg/kg body weight twice daily for the first 2 days, starting 1 day before the light exposure and once daily for the remaining 3 days. Four days after the light exposure OCT, immunohistochemical stainings, real-time RT-PCR analysis, and RNA sequencing were performed to determine the in vivo effects of minocycline treatment (schematic drawing). Representative photomicrographs show retinal sections (**a**–**c**) and flat mounts (**d**–**f**) stained with Iba1 (*green*), TSPO (*red*) (**g**–**i**), and GFAP (*red*) (**j**–**l**). In control retinas, microglial cells were located in the OPL, IPL, and GCL (**a**, **d**); low levels of TSPO were only observable in the GCL (**g**); and signs for Müller cell gliosis were not detectable (**j**). **b**, **e**, **h**, and **k** Photomicrographs of light-exposed mice showed a massive thinning of the ONL and an increased number of amoeboid shaped, reactive microglia in the ONL, and the subretinal space (**b**, **e**). **h** A strong up-regulation of TSPO expression was detectable in microglia. **k** Up-regulation of GFAP indicates Müller cell gliosis. **c**, **f**, **i**, and **l** Representative photomicrographs of minocycline-treated mice 4 days after light exposure. Compared to untreated controls, the ONL of minocycline-treated mice appeared markedly preserved and less microglia were detectable in the ONL and the subretinal space (**c**, **f**). **i** Microglial cells were negative for TSPO expression. **l** GFAP expression was only detectable in astrocytes, indicating a lack of reactive Müller cells. *TSPO* translocator protein, *GFAP* glial fibrillary acid protein, *ONL* outer nuclear layer, *OPL* outer plexiform layer, *INL* inner nuclear layer, *IPL* inner plexiform layer, *GCL* ganglion cell layer. Scale bar 50 μm

To test the effects of minocycline on microglial responses in the damaged retina in vivo, we selected the murine model of acute light-induced degeneration. This model mimics several features of AMD including immune activation and apoptosis of photoreceptor cells. After 16 h of dark adaptation, BALB/cJ mice were exposed to white light with an intensity of 15.000 lux for 1 h. The mice received daily intraperitoneal injections of 45 mg/kg minocycline, starting 1 day before the light exposure for five consecutive days. The first 2 days, the mice received two injections per day and then once daily for the remaining 3 days (Fig. 3, top, schematic drawing). Four days after light exposure, the effect of minocycline administration was analyzed by staining of retinal sections and flat mounts with markers for reactive gliosis. In control animals, retinal immunolabeling with the marker Iba1 showed ramified microglia in the plexiform layers as expected (Fig. 3a). Light exposure caused a severe thinning of the outer nuclear layer and many amoeboid shaped microglia appeared in the degenerating photoreceptor layer and the subretinal area (Fig. 3b). This prominent degeneration of photoreceptors and accumulation of microglia in the outer retina was not detectable in retinas of mice treated with minocycline (Fig. 3c). To better analyze the microglial network and localization, retinal flat mounts were stained with Iba1. In contrast to healthy controls, where microglial cells are absent in the outer retina, retinal flat mounts from light-exposed mice showed high numbers of amoeboid shaped microglia in the subretinal area (Fig. 3d, e). Treatment with minocycline clearly prevented migration of many microglia to the subretinal area, and the cells in this region had a more ramified morphology (Fig. 3f).

To better characterize the reactive phenotype of glia cells, the expression of the activation markers TSPO for microglia and glial fibrillary acid protein (GFAP) for Müller cells and astrocytes were analyzed. Exposure to light initiated a strong increase in TSPO expression, especially confined to outer retinal microglia (Fig. 3h). In contrast, administration of minocycline strongly reduced TSPO expression, indicating a less reactive microglia phenotype (Fig. 3i). Expression of GFAP in Müller cells and astrocytes of retinas exposed to light was also increased compared to controls (Fig. 3j, k), whereas minocycline-treated animals displayed only weak GFAP staining (Fig. 3l). These findings indicate that the reactive gliosis elicited by acute light damage can be significantly suppressed by minocycline treatment.

### Minocycline counter-regulates pro-inflammatory gene expression in retinal degeneration

**Fig. 4:**
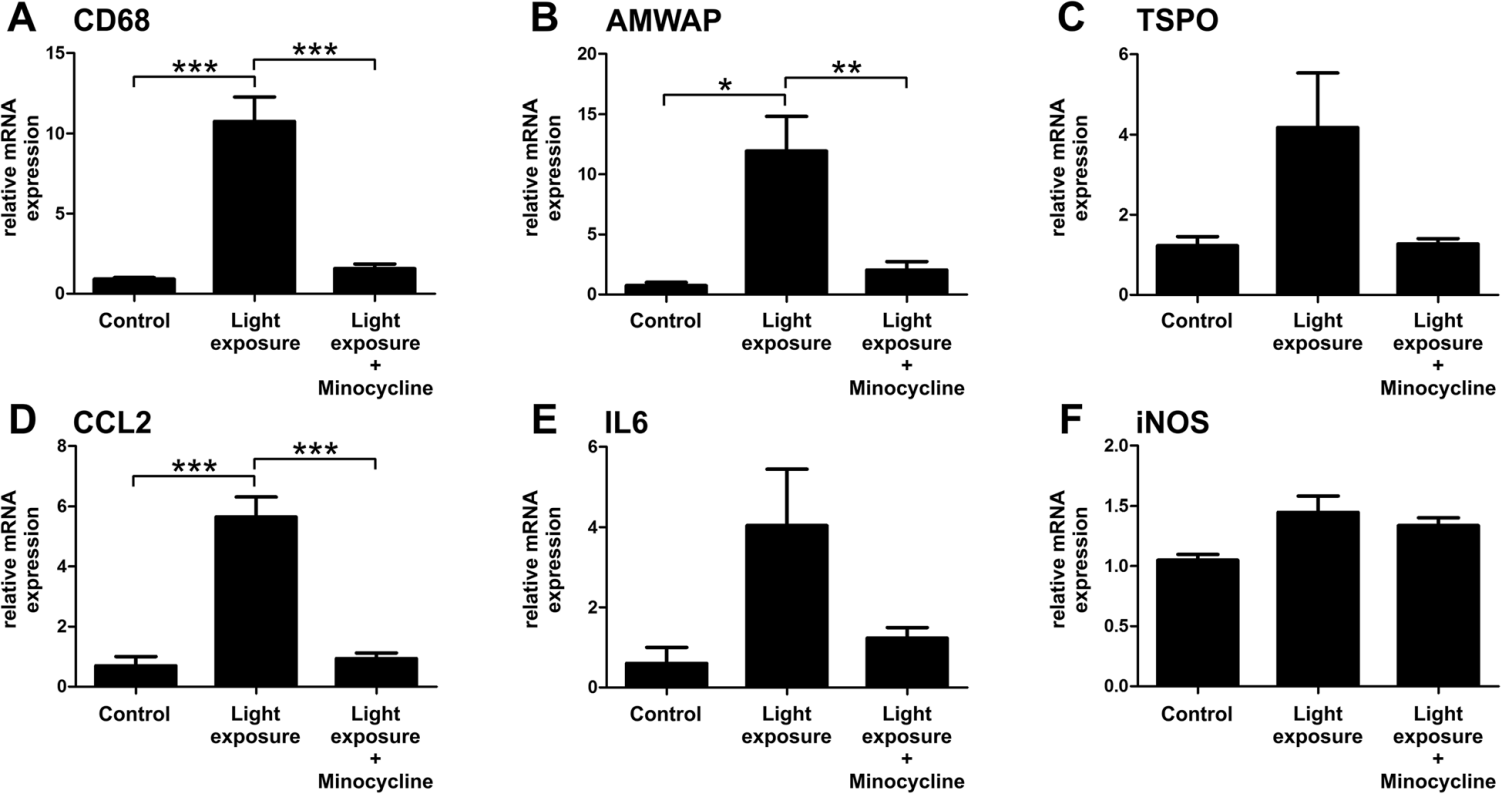
Minocycline treatment dampens microglia-related pro-inflammatory marker expression after light exposure. To determine the mRNA expression of inflammation associated genes in the retina, qRT-PCR analysis were performed 4 days after light exposure. **a**–**c** The microglia activation markers CD68 and AMWAP were significantly up-regulated after light exposure, and TSPO levels were also increased. In contrast, retinas from minocycline-treated mice expressed significantly less CD68 and AMWAP transcripts and also reduced mRNA levels of TSPO. **d** Minocycline treatment reduced the expression of CCL2 that was significantly elevated by light exposure. **e** IL6 was enhanced by light exposure, which was reversed in the minocycline administration group. **f** iNOS expression was not significantly altered. Data show mean ± SEM (control *n* = 4, light exposure *n* = 4, light exposure and minocycline treatment *n* = 5/group, measured in triplicates) with *p < 0.05, **p < 0.01, ***p < 0.001

In addition to these in situ analyses of retinas, we investigated whether mRNA levels of microglia-related pro-inflammatory marker genes were influenced by minocycline administration in vivo. CD68, AMWAP, and TSPO are markers that are associated with microglia proliferation and reactivity [38–41]. Four days after light exposure all three markers were clearly up-regulated in the retina, with especially high induction levels of CD68 and AMWAP (Fig. 4a–c). In mice treated with minocycline, the expression of CD68 (Fig. 4a, *p* = 0.0002) and AMWAP (Fig. 4b, *p* = 0.007) were significantly suppressed and mRNA levels of TSPO were reduced (Fig. 4c, *p* = 0.057). Furthermore, transcripts that reflect the activation of key microglial pathways including chemotaxis, pro-inflammatory cytokines, and radical production were analyzed. In retinas of light exposed mice that were treated with minocycline, mRNA levels of the chemotactic molecule CCL2 were significantly decreased compared to untreated animals (Fig. 4d, *p* < 0.0001). The expression of IL6 was also elevated after exposure to light and returned to baseline in the minocycline administration group (Fig. 4e). In contrast to our in vitro data, no significant differences in iNOS expression were detectable after light exposure or minocycline treatment after the 4-day period (Fig. 4f). This suggests that minocycline also exerts microglia-related anti-inflammatory effects in vivo in the retina.

### Minocycline dampens early microglia responses to acute white light

**Fig. 5:**
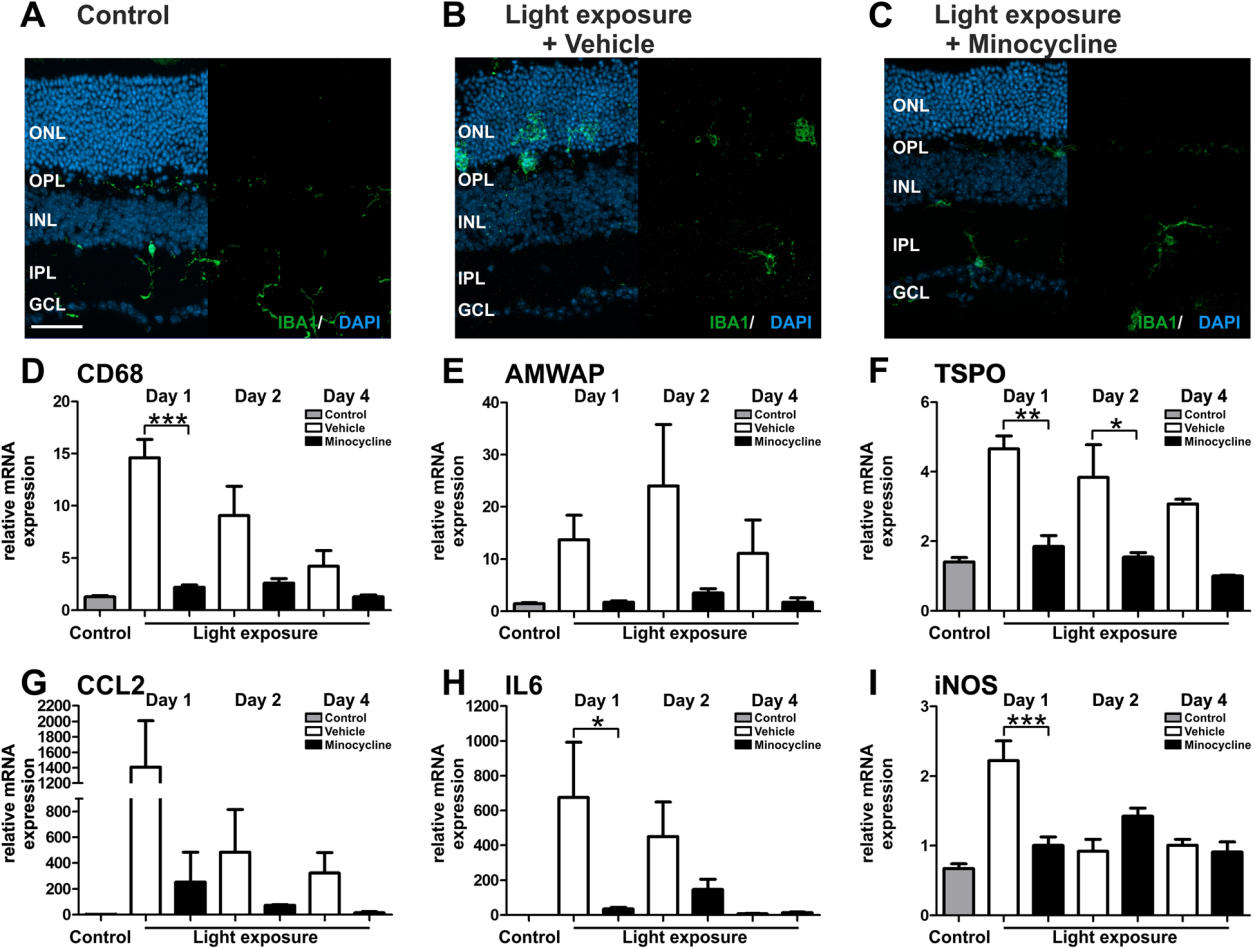
Minocycline suppresses early microglial activation after exposure to bright white light. Representative photomicrographs show retinal sections (**a**–**c**) stained with Iba1 (*green*) of control, vehicle- or minocycline-treated mice 1 day after light exposure. In healthy controls, microglial cells are only located in the plexiform layers and GCL (**a**). Images of light-exposed vehicle-treated mice show amoeboid microglia located in the ONL, whereas retinal structures appear unaffected 1 day after light exposure (**b**). Compared to vehicle-treated mice, amoeboid, reactive microglia are absent in the ONL of mice treated with minocycline (**c**). *ONL* outer nuclear layer, *OPL* outer plexiform layer, *INL* inner nuclear layer, *IPL* inner plexiform layer, *GCL* ganglion cell layer. Scale bar 50 μm. Data show representative photomicrographs (control *n* = 6, light exposure and vehicle treatment *n* = 3, light exposure and minocycline treatment *n* = 3/group). Retinal mRNA expression of microglia and inflammation-associated genes were determined by real-time RT-PCR at 1, 2, or 4 days after light exposure (**d**–**i**). Compared to controls, the microglial-associated markers CD68, AMWAP, and TSPO were markedly increased in vehicle-treated mice peaking 1 day after light exposure. Expression levels in minocycline-treated mice were lower at every analyzed time point (**d**–**f**). CCL2 was induced in light-exposed vehicle-treated mice, which was suppressed in the minocycline-treated group (**g**). Minocycline treatment also significantly reduced the expression of IL6 and iNOS, which was strongly upregulated in the vehicle-treated group (**h**, **i**). Data show mean ± SEM (control *n* = 6, light exposure and vehicle treatment *n* = 3, light exposure and minocycline treatment *n* = 3/group, measured in triplicates) with *p < 0.05, **p < 0.01, ***p < 0.001

Analysis 4 days after light exposure revealed that minocycline reduced the number of activated microglial cells and impaired their pro-inflammatory gene transcription. We next studied whether minocycline also affects early microglia responses at time points that lack overt cell death as trigger for migration and reactivity. Furthermore, we included a vehicle-treated control group in these experiments to exclude potentially protective stress-related effects. One day after light exposure retinal sections from control, vehicle- or minocycline-treated mice were stained with Iba1. Ramified microglia were present in the plexiform layers and the ganglion cell layer of control animals (Fig. 5a). In contrast, retinas from light-exposed and vehicle-treated mice revealed many amoeboid shaped microglia in the outer nuclear layer (ONL), which did not show structural changes at this early time point after light challenge (Fig. 5b). Minocycline treatment completely prevented this early microglia migration into the ONL (Fig. 5c).

We next also analyzed the mRNA expression of microglia-associated markers in a time kinetic experiment. Light exposure enhanced the expression of the CD68, AMWAP, and TSPO in vehicle-treated mice already 1 day after exposure to white light (Fig. 5d–f). Minocycline administration dampened the expression of these genes at every analyzed time point and reached statistical significance for CD68 (Fig. 5d, vehicle versus control *p* < 0.0001, vehicle versus minocycline *p* = 0.0001 1 day after light exposure) and TSPO (Fig. 5f, vehicle versus control *p* = < 0.0001, vehicle versus minocycline *p* = 0.0018 1 day after light exposure, and vehicle versus minocycline *p* = 0.0113 2 days after light exposure). The migration marker CCL2 was strongly up-regulated in light-exposed vehicle controls, reaching statistical significance 1 day after light exposure (control versus vehicle *p* = 0.0046). Minocycline-treated mice expressed lower CCL2 at every analyzed time point. Furthermore, minocycline also significantly reduced the expression of IL6, which was strongly upregulated in the vehicle-treated group (Fig. 5h, control versus vehicle *p* = 0.0082, vehicle versus minocycline *p* = 0.0371 1 day after light exposure). In contrast to our previous analysis after 4 days (Fig. 4f), iNOS was induced 1 day after light exposure in vehicle controls and was significantly reduced in the minocycline administration group (Fig. 5i, control versus vehicle *p* < 0.0001, vehicle versus minocycline *p* = 0.0004 1 day after light exposure).

In a small pilot experiment, we further explored whether microglia may be even earlier alarmed in the light damage condition. Indeed, ramified microglia projected their long protrusions reaching through the ONL already 4 h after light exposure (Additional file 1: Figure S1A, B). mRNA analysis in these retinas clearly showed increased expression of CCL2, IL6, and CD68 in the absence of changes in caspase 8 levels (Additional file 1: Figure S1C–F). Therefore, we conclude that microglial activation is an early event after light exposure that is not only triggered by dead cells and that minocycline is very effective in attenuating this process.

### Minocycline exerts global anti-inflammatory effects on the retinal transcriptome

**Fig. 6:**
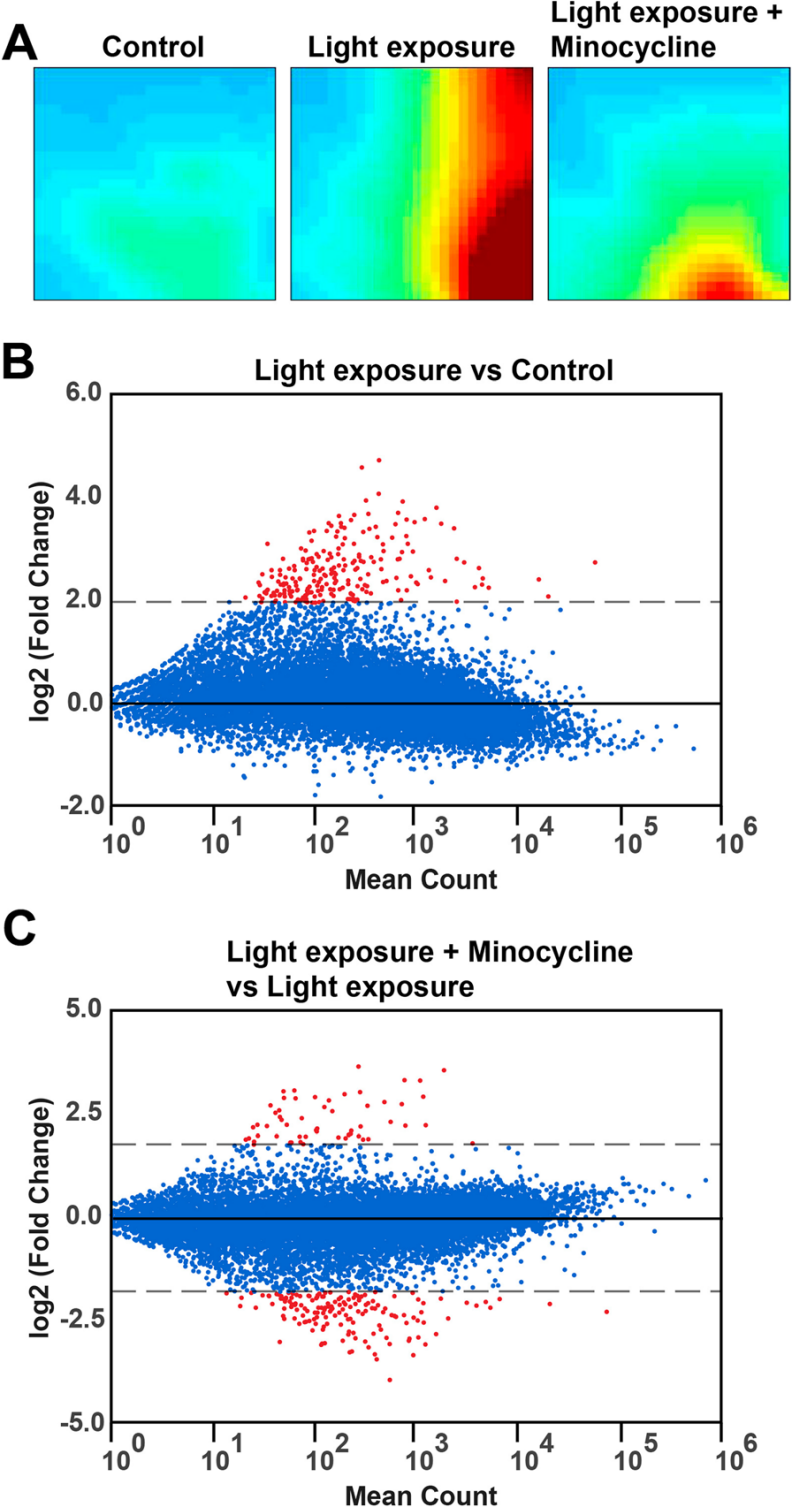
Light exposure and minocycline exert global effects on the retinal transcriptome. **a** Gene Expression Dynamics Inspector (GEDI) analysis of the complete RNA-seq dataset (Gene Expression Omnibus Series Nr. GSE71025) from control retinas and light-exposed retinas in the absence or presence of minocycline. Biological duplicates were analyzed for each condition and the pseudo-color scale indicates high (*red*) or low (*blue*) expression levels. **b**, **c** DESeq2 analysis to determine differential gene expression between the indicated groups using a log2 fold change cutoff of ≥2 and ≤−2. The MA-plots demonstrate that light exposure caused a global induction of 217 genes (**b**), whereas light exposure plus minocycline administration reversed this effect with many counter-regulated transcripts (**c**)

We then asked whether minocycline treatment has a global effect on gene expression in the light exposure model of retinal degeneration. RNA-sequencing analysis with two biological replicates was performed on total retinal RNA isolated from control mice, from animals after light exposure, and from light-exposed animals that received minocycline. We first used the Gene Expression Dynamics Inspector (GEDI) to determine the global patterns of gene expression in the three different conditions. GEDI is based on self-organizing maps to identify genome-wide transcriptome activity via “gestalt” recognition [37]. GEDI is sample-oriented rather than gene-oriented, which allows the identification of genome-wide patterns. Each mosaic tile in the GEDI map represents a gene cluster that is expressed at similar levels, with blue color indicating a low level and red corresponding to high expression. The three GEDI maps show a highly dynamic regulation of gene expression in light-exposed and minocycline-treated retinas compared to control (Fig. 6a). Light exposure initiated the formation of a prominent red cluster band that was nearly reversed by minocycline treatment (Fig. 6a). In the next step, we performed DESeq2 analysis to determine differential gene expression between the groups using a log2 fold change cutoff of ≥2. The MA-plots demonstrate that light exposure caused a significant induction of 217 genes with no down-regulated transcripts (Fig. 6b). In contrast, the light-exposed versus light-exposed plus minocycline treatment analysis identified 113 down-regulated and 38 up-regulated transcripts (Fig. 6c). A closer look at the differentially expressed genes showed that most of the transcripts induced under light damage conditions belong to immune-related biological pathways including markers directly related to microglia reactivity such as macrophage scavenger receptor 1 (MSR1), AMWAP (alias WFDC17), CD68, and complement factor C3 (Additional file 2: Table S1). The vast majority of transcripts down-regulated by minocycline treatment was induced by light exposure alone, and hence, their expression was reversed (Additional file 3: Table S2). These results demonstrate that both light damage and minocycline have a major impact on the global pattern of gene expression in the retina and that minocycline administration effectively counterbalances most of the pro-inflammatory signaling events in the degenerating retina.

### Minocycline treatment protects the retina from light-induced degeneration

**Fig. 7:**
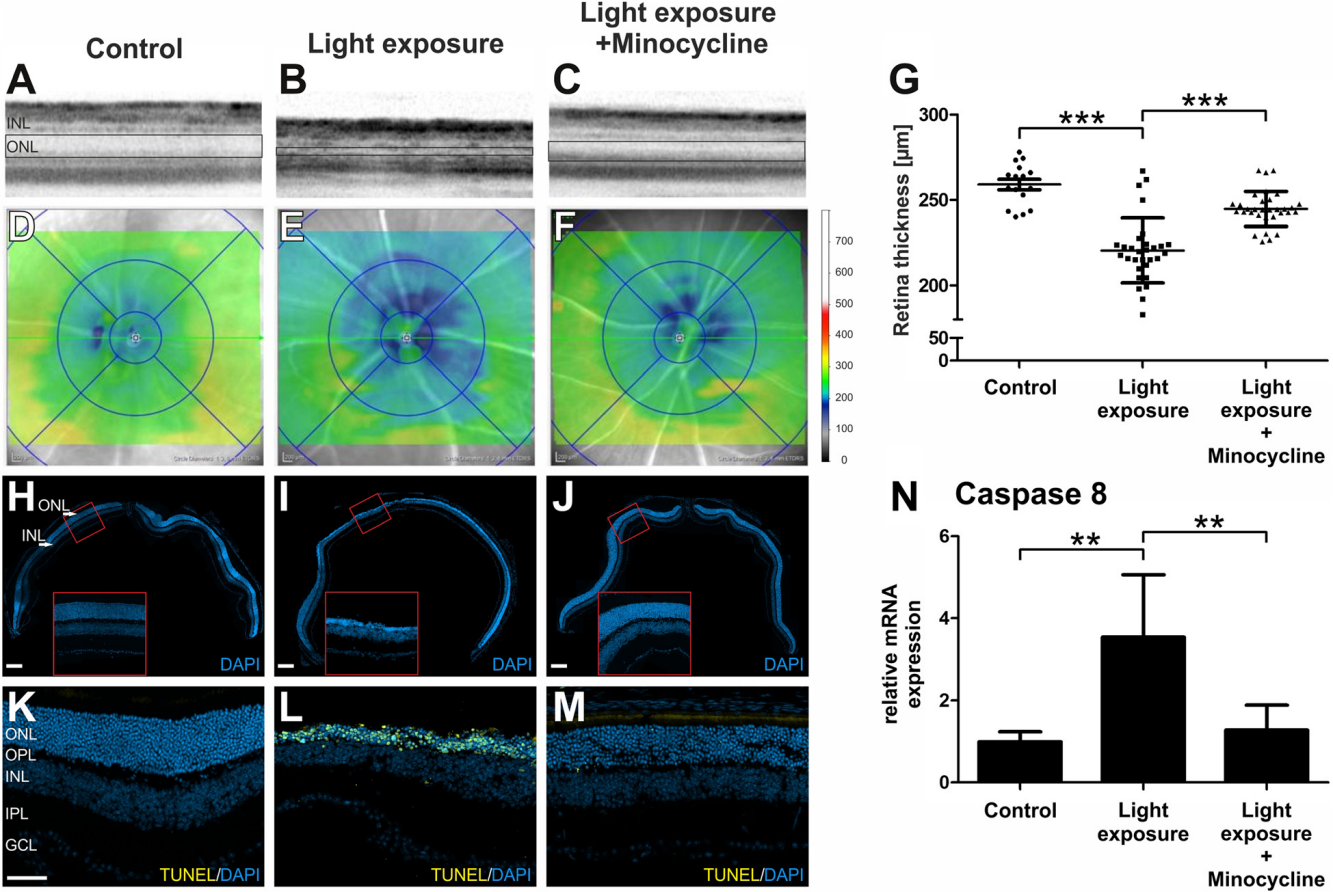
Minocycline prevents light-induced retinal degeneration. **a**–**g** SD-OCT was performed 4 days after light exposure to analyze changes in retinal structures. **a**–**c** Light-exposed mice show an altered reflectance in the ONL, which was not present in minocycline-treated mice. **d**–**f** Representative heat maps show the average retinal thickness of control, light-exposed minocycline-treated and untreated mice. Light-exposed mice show a significant thinning of the retina, especially in the central area, which was preserved by minocycline treatment. **g** One data point represents the average thickness of the central retina [μm], calculated from four different areas around the optic nerve head. Data show mean ± SEM (control *n* = 16 eyes, light exposure *n* = 30 eyes, light exposure and minocycline treatment *n* = 32 eyes). **h**–**j** Representative panorama sections of the retina were stained with DAPI to further characterize structural changes in the retina. Light exposure caused severe degeneration particularly of the ONL. Minocycline administration reduced degeneration, represented by a clearly thicker photoreceptor layer, scale bar, 200 μM. **k**–**m** Representative photomicrographs of TUNEL-stained retinal sections show the amount of apoptotic cells 4 days after light exposure. Light exposure caused a strong increase of apoptotic cells, especially in the ONL. Less TUNEL-positive cells were visible after minocycline administration. **n** RNA analyses revealed a significantly higher expression of caspase 8 in light-exposed retinas, compared to retinas of minocycline-treated mice, indicating a higher amount of apoptotic cells. Scale bar, 50 μm. Data show mean ± SEM (control *n* = 4 retinas, light exposure *n* = 4 retinas, light exposure and minocycline treatment *n* = 5 retinas, measured in triplicates) *p < 0.05, **p < 0.01, ***p < 0.001

We finally asked whether targeting retinal microglia with minocycline also improves the outcome of disease progression. We first performed in vivo optical coherence tomography (OCT) of mice to detect structural changes of the retina after light exposure and under conditions of minocycline treatment. The OCT images showed clear changes in ONL reflectance in retinas of light-exposed animals, indicating a strong degeneration of the photoreceptor layer (Fig. 7a, b). In contrast, minocycline-treated mice displayed a normal hyperreflective photoreceptor layer similar to that of controls (Fig. 7c). Volume scans revealed a severe thinning of the retina, especially in the central area around the optic nerve head after light exposure, which was not observed in the minocycline-treated groups (Fig. 7d–f). Quantification of the retinal thickness in all analyzed animals demonstrate a significant reduction in the central area after light exposure (*p* < 0.0001), which could be rescued by treatment with minocycline (*p* < 0.0001) (Fig. 7g). To confirm these findings, panorama images of retinal sections proceeding through the optic nerve head were stained with DAPI. The photoreceptor layer showed a clear thinning in the group of mice exposed to light, which was not evident in mice after minocycline treatment (Fig. 7h–j, Fig. 3a–c). TUNEL stainings and mRNA analyses of caspase 8 levels also revealed that light exposure significantly induced cell death in mouse retinas, which was prevented in minocycline-treated animals (Fig. 7k–n). These data clearly point toward a strong neuroprotective effect of minocycline in conditions of acute light damage.

## Discussion

Several studies revealed that minocycline exerts anti-microbial, anti-inflammatory, anti-apoptotic, and neuroprotective properties in different animal models of neuronal degenerative diseases, including Parkinson’s disease, multiple sclerosis, Alzheimer’s disease, Huntington’s disease, amyotrophic lateral sclerosis, and retinitis pigmentosa [16, 18–20, 23, 25, 42–44]. Here, we report for the first time that minocycline administration preserves the retinal structure in a white light-induced degeneration model, which mimics several features of dry AMD and monogenic retinal dystrophies. The main questions discussed in this study were if and how minocycline stimulation modulates the neurotoxic potential of microglia and whether minocycline administration could be a suitable immuno-modulatory strategy to treat retinal degeneration.

Our in vitro studies in LPS-activated BV-2 microglia demonstrated that minocycline was capable to diminish gene expression of the pro-inflammatory markers CCL2, IL6, and iNOS in a rapid temporal response. This is consistent with earlier reports with microglia isolated from rat retinas where minocycline dampened mRNA expression and secretion of IL1β, TNFα, and iNOS [45]. In line with this, Henry et al. reported that minocycline reduced mRNA levels of TLR 2, MHC-II, IL1β, and IL6 in BV-2 cells [46]. These results together with our data suggest that minocycline is highly effective in reducing pro-inflammatory gene transcription in microglia.

The production of toxic nitric oxide by phagocytes plays an important role in degenerative processes [47–49]. Here, we demonstrated that minocycline effectively dampened nitric oxide production of LPS-stimulated microglia. Furthermore, minocycline reduced the general neurotoxic potential of reactive microglia on 661W photoreceptor cells. Supernatants from microglia that were stimulated with both LPS and minocycline were less toxic to photoreceptors than those of cells that were treated with LPS only. Investigations in RAW264.7 macrophages and spinal cord microglia also confirmed that minocycline blocks the production of toxic nitric oxide [50, 51]. Hence, others have found an association of reduced iNOS levels and NO itself with delayed neurodegeneration [19, 47, 52]. In the 1-methyl-4-phenyl-1,2,3,6-tetrahydropyridine (MPTP) mouse model of Parkinson’s disease, minocycline reduced MPP+ induced glial iNOS expression, which was most likely due to an inhibition of p38 MAPK phosphorylation [19].

Besides the reduction of NO production, several other mechanisms of action are discussed for the beneficial effects of minocycline. Indeed, we here also showed that minocycline administration directly protected photoreceptors from microglia mediated death. In a model of Huntington’s disease, minocycline delayed disease progression and mortality by inhibition of caspase-1 and caspase-3 up-regulation, enzymes which play an important role in the induction of necrosis and apoptosis [43]. Studies in R6/2 mice confirmed that minocycline can inhibit both caspase-independent (apoptosis inducing factor) and caspase-dependent (Smac/Diablo and cytochrome c) mitochondrial cell death pathways [53]. However, despite its ability to reduce pro-inflammatory markers in a mouse model for age-related neuron loss, minocycline failed to inhibit apoptosis and neuron loss [54].

A further mechanism how minocycline exerts its protective functions is the inhibition of matrix metalloproteases (MMPs). MMPs are a family of zinc- and calcium-dependent proteolytic enzymes that are responsible for the degradation of structural proteins in the extracellular matrix and their activation is associated with neurological disorders. Thus, blockage of MMPs by minocycline decreased the cerebral infarct size in a mouse model of focal cerebral ischemia. This effect was most likely due to the inhibition of CCL2, TNFα, and indoleamine 2,3-dioxygenase (IDO) expression [55, 56].

Our OCT images and immunohistochemical analysis showed that the treatment with minocycline preserved retinal structure and reduced the amount of apoptotic cells after exposure to bright white light. The protection of photoreceptors was associated with a reduced number of reactive, amoeboid-shaped, TSPO-positive microglia in the outer retina. Accordingly, our mRNA analyses of retinas revealed suppression of microglial activation markers including AMWAP and CD68 after administration of minocycline [38–40]. Analysis at different time points after light exposure revealed that microglial activation is an early event that precedes photoreceptor death. Moreover, our RNA-sequencing data revealed a global transcriptomic effect of minocycline with a complete cluster of light-damage-induced pro-inflammatory genes that was counter-regulated by minocycline. Of note, the RNA-seq dataset also detected up-regulation of structural retinal genes such as keratins and adhesion molecules by minocycline. These results strongly indicate that modulation of microglia reactivity is a key mechanism in minocycline’s mode of action in the retina. This notion is supported by studies demonstrating that inhibition of microglia with minocycline protects from neuronal degenerative diseases including Alzheimer disease or Parkinson disease [16, 18]. In the eye, minocycline could also reduce microglial activation and improve neuronal function [13, 25, 57, 58]. In a model of green light exposure, preservation of retinal structure and ERG amplitudes by minocycline was associated with reduced numbers of CD11b+ cells in the outer retina [58]. To mimic exposure to bright daylight, which is discussed as a contributing factor for retinal degenerations, we used 15.000 lux UV-free white light in our studies. White light has an emission spectrum similar to that of daylight, and it is less artificial than light of a particular wavelength [59, 60]. Furthermore, white light contains also short wavelength blue light (403 nm) which is thought to have a higher damaging potential than light of longer wavelength including green light (490–580 nm), which was used in earlier studies [58]. The higher damaging potential of blue light is due to a process called photo-reversal, the regeneration of rhodopsin from bleaching intermediates that results in a higher number of photon absorption in a certain time span. Because of different light exposure settings in our study and the work of Zhang et al. [58], different underlying damage mechanisms cannot be ruled out [28, 59, 61, 62].

In the *rd10* mouse model for retinitis pigmentosa, minocycline inhibited microglial activation and down-regulated the expression of pro-inflammatory molecules including TNFα, COX1, and COX2. Moreover, pro-apoptotic molecules such as BAX and Caspase 3 were suppressed by minocycline, and the retinal structure and function were preserved [25]. Of note, minocycline could also diminish photoreceptor death in *rds* mice by a microglia-independent mechanism as depletion of microglia by clodronate prevented their recruitment but failed to inhibit photoreceptor apoptosis [63].

## Conclusions

We have shown that microglia activity is an early event in retinal degeneration induced by bright white light. Minocycline administration preserved the retinal structure by a global counter-regulation of microglial pro-inflammatory responses and direct effects on photoreceptors during light exposure. Our data suggest that treatment strategies that modulate microglial reactivity and support photoreceptor survival provide a potential therapeutic option for retinal degenerative diseases.

## Additional files

Additional file 1: Figure S1. Early microglial activation after exposure to bright white light. Representative photomicrographs show retinal sections (A–B) stained with Iba1 (green) of control mice and animals 4 h after light exposure. In healthy controls, microglial cells are only located in the plexiform layers and GCL (A). Images of light exposed mice show microglia with protrusions in the ONL (B). ONL, outer nuclear layer; OPL, outer plexiform layer; INL, inner nuclear layer; IPL, inner plexiform layer; GCL, ganglion cell layer. Scale bar 50 μm. Data show representative photomicrographs (control *n* = 5, light exposure *n* = 4). Retinal mRNA expression of microglia-associated genes and caspase 8 were determined by real-time RT-PCR (C-F). Data show mean ± SEM (control *n* = 5 retinas, light exposure *n* = 4 retinas measured in triplicates) with **p* < 0.05. (JPG 684 kb) Additional file 2: Table S1. Differentially expressed transcripts comparing light exposure versus control. (DOCX 85 kb) Additional file 3: Table S2. Differentially expressed transcripts comparing light exposure versus light exposed plus minocyline treatment. (DOCX 80 kb)

## References

[1] Wong WL, Su X, Li X, Cheung CM, Klein R, Cheng CY (2014). Global prevalence of age-related macular degeneration and disease burden projection for 2020 and 2040: a systematic review and meta-analysis. Lancet Glob Health.

[2] Jager RD, Mieler WF, Miller JW (2008). Age-related macular degeneration. N Engl J Med.

[3] Xu H, Chen M, Forrester JV (2009). Para-inflammation in the aging retina. Prog Retin Eye Res.

[4] Gupta N, Brown KE, Milam AH (2003). Activated microglia in human retinitis pigmentosa, late-onset retinal degeneration, and age-related macular degeneration. Exp Eye Res.

[5] Karlstetter M, Scholz R, Rutar M, Wong WT, Provis JM, Langmann T (2015). Retinal microglia: just bystander or target for therapy?. Prog Retin Eye Res.

[6] Hume DA, Perry VH, Gordon S (1983). Immunohistochemical localization of a macrophage-specific antigen in developing mouse retina: phagocytosis of dying neurons and differentiation of microglial cells to form a regular array in the plexiform layers. J Cell Biol.

[7] Kettenmann H, Hanisch UK, Noda M, Verkhratsky A (2011). Physiology of microglia. Physiol Rev.

[8] Damani MR, Zhao L, Fontainhas AM, Amaral J, Fariss RN, Wong WT (2011). Age-related alterations in the dynamic behavior of microglia. Aging Cell.

[9] Nimmerjahn A, Kirchhoff F, Helmchen F (2005). Resting microglial cells are highly dynamic surveillants of brain parenchyma in vivo. Science.

[10] Aloisi F (2001). Immune function of microglia. Glia.

[11] Langmann T (2007). Microglia activation in retinal degeneration. J Leukoc Biol.

[12] Karlstetter M, Langmann T (2014). Microglia in the aging retina. Adv Exp Med Biol.

[13] Zhao L, Zabel MK, Wang X, Ma W, Shah P, Fariss RN (2015). Microglial phagocytosis of living photoreceptors contributes to inherited retinal degeneration. EMBO Mol Med.

[14] Sierra A, Gottfried-Blackmore AC, McEwen BS, Bulloch K (2007). Microglia derived from aging mice exhibit an altered inflammatory profile. Glia.

[15] Roque RS, Rosales AA, Jingjing L, Agarwal N, Al-Ubaidi MR (1999). Retina-derived microglial cells induce photoreceptor cell death in vitro. Brain Res.

[16] Wu DC, Jackson-Lewis V, Vila M, Tieu K, Teismann P, Vadseth C (2002). Blockade of microglial activation is neuroprotective in the 1-methyl-4-phenyl-1,2,3,6-tetrahydropyridine mouse model of Parkinson disease. J Neurosci.

[17] Amor S, Puentes F, Baker D, van der Valk P (2010). Inflammation in neurodegenerative diseases. Immunology.

[18] Biscaro B, Lindvall O, Tesco G, Ekdahl CT, Nitsch RM (2012). Inhibition of microglial activation protects hippocampal neurogenesis and improves cognitive deficits in a transgenic mouse model for Alzheimer’s disease. Neurodegener Dis.

[19] Du Y, Ma Z, Lin S, Dodel RC, Gao F, Bales KR (2001). Minocycline prevents nigrostriatal dopaminergic neurodegeneration in the MPTP model of Parkinson’s disease. Proc Natl Acad Sci U S A.

[20] Popovic N, Schubart A, Goetz BD, Zhang SC, Linington C, Duncan ID (2002). Inhibition of autoimmune encephalomyelitis by a tetracycline. Ann Neurol.

[21] Owolabi SA, Saab CY (2006). Fractalkine and minocycline alter neuronal activity in the spinal cord dorsal horn. FEBS Lett.

[22] Mika J, Osikowicz M, Makuch W, Przewlocka B (2007). Minocycline and pentoxifylline attenuate allodynia and hyperalgesia and potentiate the effects of morphine in rat and mouse models of neuropathic pain. Eur J Pharmacol.

[23] Garrido-Mesa N, Zarzuelo A, Galvez J (2013). Minocycline: far beyond an antibiotic. Br J Pharmacol.

[24] Niimi N, Kohyama K, Matsumoto Y (2013). Minocycline suppresses experimental autoimmune encephalomyelitis by increasing tissue inhibitors of metalloproteinases. Neuropathology.

[25] Peng B, Xiao J, Wang K, So KF, Tipoe GL, Lin B (2014). Suppression of microglial activation is neuroprotective in a mouse model of human retinitis pigmentosa. J Neurosci.

[26] Cruickshanks KJ, Klein R, Klein BE (1993). Sunlight and age-related macular degeneration: the beaver dam eye study. Arch Ophthalmol.

[27] Swaroop A, Chew EY, Rickman CB, Abecasis GR (2009). Unraveling a multifactorial late-onset disease: from genetic susceptibility to disease mechanisms for age-related macular degeneration. Annu Rev Genomics Hum Genet.

[28] Grimm C, Reme CE (2013). Light damage as a model of retinal degeneration. Methods Mol Biol.

[29] Marc RE, Jones BW, Watt CB, Vazquez-Chona F, Vaughan DK, Organisciak DT (2008). Extreme retinal remodeling triggered by light damage: implications for age related macular degeneration. Mol Vis.

[30] Narimatsu T, Ozawa Y, Miyake S, Kubota S, Hirasawa M, Nagai N (2013). Disruption of cell-cell junctions and induction of pathological cytokines in the retinal pigment epithelium of light-exposed mice. Invest Ophthalmol Vis Sci.

[31] Pennesi ME, Neuringer M, Courtney RJ (2012). Animal models of age related macular degeneration. Mol Aspects Med.

[32] Blasi E, Barluzzi R, Bocchini V, Mazzolla R, Bistoni F (1990). Immortalization of murine microglial cells by a v-raf/v-myc carrying retrovirus. J Neuroimmunol.

[33] Ebert S, Weigelt K, Walczak Y, Drobnik W, Mauerer R, Hume DA (2009). Docosahexaenoic acid attenuates microglial activation and delays early retinal degeneration. J Neurochem.

[34] Ebert S, Schoeberl T, Walczak Y, Stoecker K, Stempfl T, Moehle C (2008). Chondroitin sulfate disaccharide stimulates microglia to adopt a novel regulatory phenotype. J Leukoc Biol.

[35] Dobin A, Davis CA, Schlesinger F, Drenkow J, Zaleski C, Jha S (2013). STAR: ultrafast universal RNA-seq aligner. Bioinformatics.

[36] Anders S, Huber W (2010). Differential expression analysis for sequence count data. Genome Biol.

[37] Eichler GS, Huang S, Ingber DE (2003). Gene expression dynamics inspector (GEDI): for integrative analysis of expression profiles. Bioinformatics.

[38] Karlstetter M, Walczak Y, Weigelt K, Ebert S, Van den Brulle J, Schwer H (2010). The novel activated microglia/macrophage WAP domain protein, AMWAP, acts as a counter-regulator of proinflammatory response. J Immunol.

[39] Karlstetter M, Nothdurfter C, Aslanidis A, Moeller K, Horn F, Scholz R (2014). Translocator protein (18 kDa) (TSPO) is expressed in reactive retinal microglia and modulates microglial inflammation and phagocytosis. J. Neuroinflammation.

[40] Aslanidis A, Karlstetter M, Scholz R, Fauser S, Neumann H, Fried C (2015). Activated microglia/macrophage whey acidic protein (AMWAP) inhibits NFkappaB signaling and induces a neuroprotective phenotype in microglia. J Neuroinflammation.

[41] Scholz R, Caramoy A, Bhuckory MB, Rashid K, Chen M, Xu H (2015). Targeting translocator protein (18 kDa) (TSPO) dampens pro-inflammatory microglia reactivity in the retina and protects from degeneration. J Neuroinflammation.

[42] Nikodemova M, Watters JJ, Jackson SJ, Yang SK, Duncan ID (2007). Minocycline down-regulates MHC II expression in microglia and macrophages through inhibition of IRF-1 and protein kinase C (PKC)alpha/betaII. J Biol Chem.

[43] Chen M, Ona VO, Li M, Ferrante RJ, Fink KB, Zhu S (2000). Minocycline inhibits caspase-1 and caspase-3 expression and delays mortality in a transgenic mouse model of huntington disease. Nat Med.

[44] Tikka TM, Vartiainen NE, Goldsteins G, Oja SS, Andersen PM, Marklund SL (2002). Minocycline prevents neurotoxicity induced by cerebrospinal fluid from patients with motor neurone disease. Brain.

[45] Tso MO, Zhu X, Wang AL, Yu AC, Lau LT, Lee C (2005). Minocycline inhibits LPS-induced retinal microglia activation. Neurochem Int.

[46] Henry CJ, Huang Y, Wynne A, Hanke M, Himler J, Bailey MT (2008). Minocycline attenuates lipopolysaccharide (LPS)-induced neuroinflammation, sickness behavior, and anhedonia. J Neuroinflammation.

[47] Yang L-p, Li Y, Zhu X-a, Tso MOM (2007). Minocycline delayed photoreceptor death in the rds mice through iNOS-dependent mechanism. Mol Vis.

[48] Yrjanheikki J, Tikka T, Keinanen R, Goldsteins G, Chan PH, Koistinaho J (1999). A tetracycline derivative, minocycline, reduces inflammation and protects against focal cerebral ischemia with a wide therapeutic window. Proc Natl Acad Sci U S A.

[49] Hoey S, Grabowski PS, Ralston SH, Forrester JV, Liversidge J (1997). Nitric oxide accelerates the onset and increases the severity of experimental autoimmune uveoretinitis through an IFN-gamma-dependent mechanism. J Immunol.

[50] Amin AR, Patel RN, Thakker GD, Lowenstein CJ, Attur MG, Abramson SB (1997). Post-transcriptional regulation of inducible nitric oxide synthase mRNA in murine macrophages by doxycycline and chemically modified tetracyclines. FEBS Lett.

[51] Matsui T, Svensson CI, Hirata Y, Mizobata K, Hua XY, Yaksh TL (2010). Release of prostaglandin E(2) and nitric oxide from spinal microglia is dependent on activation of p38 mitogen-activated protein kinase. Anesth Analg.

[52] Beattie MS (2004). Inflammation and apoptosis: linked therapeutic targets in spinal cord injury. Trends Mol Med.

[53] Wang X, Zhu S, Drozda M, Zhang W, Stavrovskaya IG, Cattaneo E (2003). Minocycline inhibits caspase-independent and -dependent mitochondrial cell death pathways in models of huntington’s disease. Proc Natl Acad Sci U S A.

[54] Cheng S, Hou J, Zhang C, Xu C, Wang L, Zou X (2015). Minocycline reduces neuroinflammation but does not ameliorate neuron loss in a mouse model of neurodegeneration. Sci Rep.

[55] Park CH, Shin TK, Lee HY, Kim SJ, Lee WS (2011). Matrix metalloproteinase inhibitors attenuate neuroinflammation following focal cerebral ischemia in mice. Korean J Physiol Pharmacol.

[56] Koistinaho M, Malm TM, Kettunen MI, Goldsteins G, Starckx S, Kauppinen RA (2005). Minocycline protects against permanent cerebral ischemia in wild type but not in matrix metalloprotease-9-deficient mice. J Cereb Blood Flow Metab.

[57] Bosco A, Inman DM, Steele MR, Wu G, Soto I, Marsh-Armstrong N (2008). Reduced retina microglial activation and improved optic nerve integrity with minocycline treatment in the DBA/2 J mouse model of glaucoma. Invest Ophthalmol Vis Sci.

[58] Zhang C, Lei B, Lam TT, Yang F, Sinha D, Tso MO (2004). Neuroprotection of photoreceptors by minocycline in light-induced retinal degeneration. Invest Ophthalmol Vis Sci.

[59] Wenzel A, Grimm C, Samardzija M, Reme CE (2005). Molecular mechanisms of light-induced photoreceptor apoptosis and neuroprotection for retinal degeneration. Prog Retin Eye Res.

[60] Youssef PN, Sheibani N, Albert DM (2011). Retinal light toxicity. Eye (Lond).

[61] Grimm C, Wenzel A, Williams T, Rol P, Hafezi F, Reme C (2001). Rhodopsin-mediated blue-light damage to the rat retina: effect of photoreversal of bleaching. Invest Ophthalmol Vis Sci.

[62] Grimm C, Reme CE, Rol PO, Williams TP (2000). Blue light’s effects on rhodopsin: photoreversal of bleaching in living rat eyes. Invest Ophthalmol Vis Sci.

[63] Hughes EH, Schlichtenbrede FC, Murphy CC, Broderick C, van Rooijen N, Ali RR (2004). Minocycline delays photoreceptor death in the rds mouse through a microglia-independent mechanism. Exp Eye Res.

