# Supplementary figures and images for: Minocycline counter-regulates pro-inflammatory microglia responses in the retina and protects from degeneration
Source: J Neuroinflammation. 2015 Nov 17;12:209. doi: 10.1186/s12974-015-0431-4 (PMC4650866; doi:10.1186/s12974-015-0431-4)

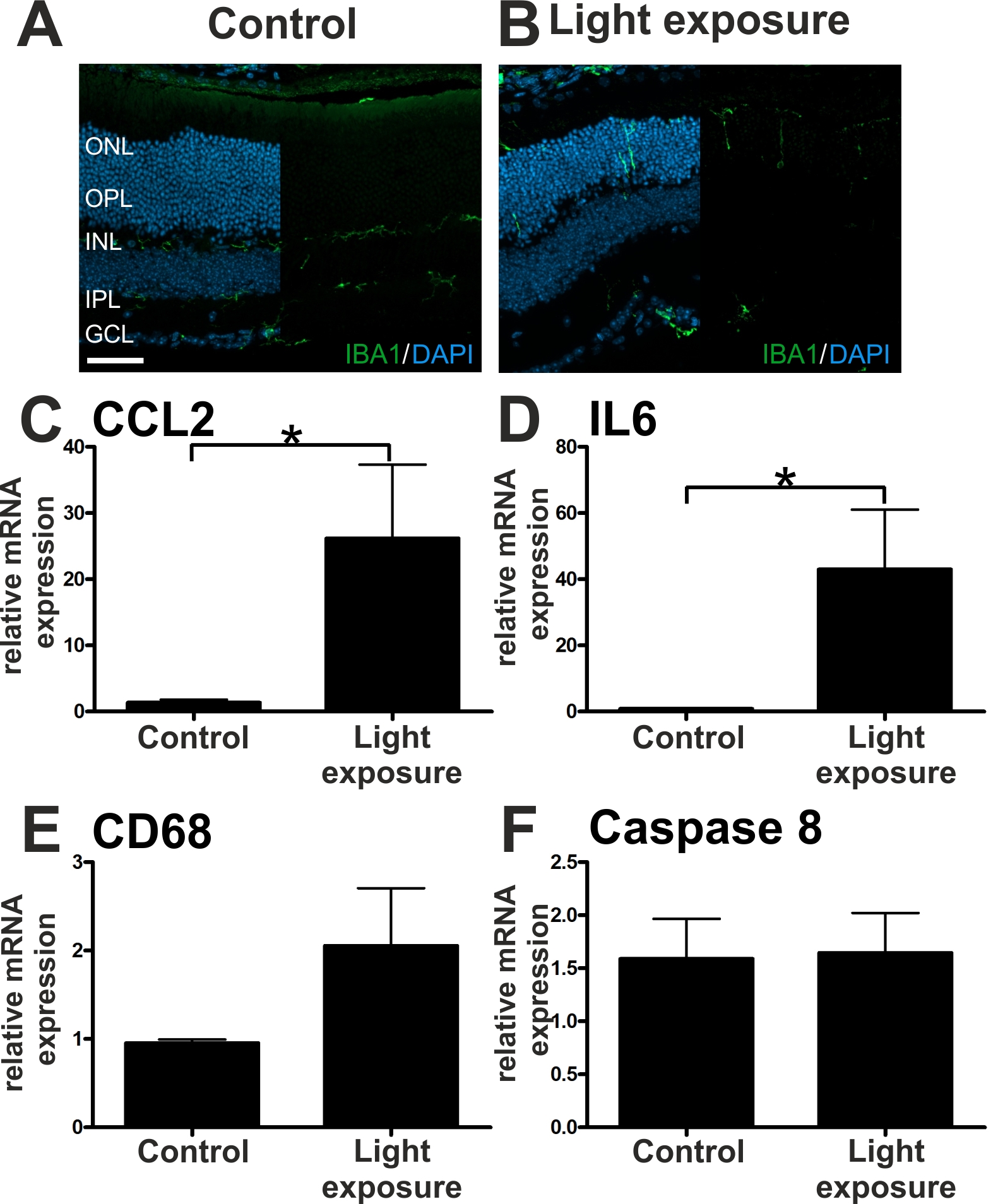

Supplement: Additional file 1: Figure S1. — Early microglial activation after exposure to bright white light. Representative photomicrographs show retinal sections (A–B) stained with Iba1 (green) of control mice and animals 4 h after light exposure. In healthy controls, microglial cells are only located in the plexiform layers and GCL (A). Images of light exposed mice show microglia with protrusions in the ONL (B). ONL, outer nuclear layer; OPL, outer plexiform layer; INL, inner nuclear layer; IPL, inner plexiform layer; GCL, ganglion cell layer. Scale bar 50 μm. Data show representative photomicrographs (control n = 5, light exposure n = 4). Retinal mRNA expression of microglia-associated genes and caspase 8 were determined by real-time RT-PCR (C-F). Data show mean ± SEM (control n = 5 retinas, light exposure n = 4 retinas measured in triplicates) with *p < 0.05. (JPG 684 kb) [file 12974_2015_431_MOESM1_ESM.jpg]
