# Supplementary material for: Minocycline counter-regulates pro-inflammatory microglia responses in the retina and protects from degeneration
Source: J Neuroinflammation. 2015 Nov 17;12:209. doi: 10.1186/s12974-015-0431-4 (PMC4650866; doi:10.1186/s12974-015-0431-4)
Supplement: Additional file 2: Table S1. — Differentially expressed transcripts comparing light exposure versus control. (DOCX 85 kb) [file 12974_2015_431_MOESM2_ESM.docx]

## Supplementary Table 1 - Differentially expressed transcripts comparing light exposure versus control

| **Nr** | **Gene** | **Mean count** | **log2 FC** |
| --- | --- | --- | --- |
| 1 | Msr1 | 281 | 4.74 |
| 2 | Ms4a6d | 213 | 3.96 |
| 3 | Clec7a | 464 | 3.94 |
| 4 | Steap4 | 195 | 4.6 |
| 5 | Slc15a3 | 125 | 3.52 |
| 6 | Timp1 | 63.9 | 3.34 |
| 7 | Cd68 | 597 | 3.54 |
| 8 | C3ar1 | 279 | 4.09 |
| 9 | Ctse | 67.1 | 3.18 |
| 10 | Hal | 70.7 | 3.37 |
| 11 | Mmp12 | 26.3 | 3.12 |
| 12 | Cd84 | 166 | 3.68 |
| 13 | Emr1 | 421 | 3.72 |
| 14 | Mki67 | 102 | 3.47 |
| 15 | Lcn2 | 434 | 2.83 |
| 16 | Lcp1 | 262 | 3.45 |
| 17 | Tlr13 | 120 | 2.7 |
| 18 | Tyrobp | 741 | 3.6 |
| 19 | Apobec1 | 202 | 2.77 |
| 20 | Siglec1 | 130 | 3.11 |
| 21 | Cybb | 116 | 3.36 |
| 22 | Top2a | 53.7 | 3.03 |
| 23 | Fcer1g | 229 | 3.41 |
| 24 | Ly9 | 69 | 2.77 |
| 25 | Cd300a | 70.5 | 2.52 |
| 26 | C3 | 224 | 3.7 |
| 27 | Cd22 | 59.1 | 2.99 |
| 28 | Wfdc17 | 41.6 | 2.64 |
| 29 | Nlrc5 | 76.9 | 2.21 |
| 30 | Trem2 | 398 | 3.5 |
| 31 | Cxcr4 | 126 | 3.45 |
| 32 | Gatm | 576 | 2.97 |
| 33 | 1300002K09Rik | 46.9 | 2.61 |
| 34 | Csf2rb2 | 50.2 | 2.04 |
| 35 | Wdfy4 | 52.2 | 2.45 |
| 36 | Laptm5 | 501 | 3.27 |
| 37 | Myo1f | 113 | 3.26 |
| 38 | Klhl6 | 56 | 2.8 |
| 39 | Cebpa | 119 | 3.03 |
| 40 | C1qc | 949 | 3.82 |
| 41 | Glipr1 | 36.6 | 2.83 |
| 42 | Plin2 | 457 | 2.96 |
| 43 | Arl11 | 60.1 | 2.63 |
| 44 | Cx3cr1 | 309 | 2.98 |
| 45 | Cdk1 | 28.9 | 2.48 |
| 46 | AF251705 | 86.7 | 2.98 |
| 47 | Arhgap25 | 44.8 | 2.45 |
| 48 | Cd53 | 371 | 3.24 |
| 49 | Nckap1l | 295 | 2.63 |
| 50 | Irf8 | 113 | 2.4 |
| 51 | Tlr7 | 155 | 3.58 |
| 52 | Ms4a6b | 67.3 | 2.84 |
| 53 | P2ry13 | 55.9 | 2.83 |
| 54 | Arhgap30 | 94.5 | 2.81 |
| 55 | Unc93b1 | 238 | 2.86 |
| 56 | Gusb | 552 | 2.49 |
| 57 | Ctsd | 27500 | 2.76 |
| 58 | Tnfrsf1b | 136 | 3.23 |
| 59 | Syk | 77.9 | 2.87 |
| 60 | Itgb2 | 165 | 3.12 |
| 61 | Bcl3 | 46.4 | 2.69 |
| 62 | Vav1 | 79.7 | 2.6 |
| 63 | Tbxas1 | 38.6 | 2.47 |
| 64 | Apoc1 | 70.9 | 2.4 |
| 65 | Fcgr2b | 103 | 2.62 |
| 66 | Fcgr3 | 201 | 3.35 |
| 67 | Itgam | 251 | 2.77 |
| 68 | Parvg | 83.1 | 2.23 |
| 69 | Sash3 | 30 | 2.17 |
| 70 | Oasl2 | 184 | 2.91 |
| 71 | Ms4a7 | 64.9 | 2.83 |
| 72 | Hexb | 2340 | 2.65 |
| 73 | Irf5 | 61.9 | 2.25 |
| 74 | Rrm2 | 16.5 | 2.08 |
| 75 | Hpgds | 118 | 2.92 |
| 76 | Tnfaip8l2 | 34.9 | 2.33 |
| 77 | Rac2 | 74.8 | 2.92 |
| 78 | Dock2 | 81.5 | 2.44 |
| 79 | Ctsz | 1710 | 2.76 |
| 80 | Gpr34 | 218 | 2.3 |
| 81 | Pik3cg | 43.5 | 2.1 |
| 82 | Anpep | 92.6 | 3.37 |
| 83 | Havcr2 | 170 | 2.62 |
| 84 | Btk | 37.2 | 2.35 |
| 85 | Galnt6 | 36.9 | 2.32 |
| 86 | Ptprc | 108 | 2.66 |
| 87 | P2ry6 | 34.3 | 2.61 |
| 88 | Xdh | 132 | 2.61 |
| 89 | Spp1 | 8340 | 2.43 |
| 90 | Casp1 | 22.4 | 2.32 |
| 91 | Cd33 | 75.8 | 2.31 |
| 92 | Rasal3 | 38 | 2.1 |
| 93 | Serpina3n | 504 | 3.59 |
| 94 | Ly86 | 178 | 2.3 |
| 95 | Crym | 1460 | 2 |
| 96 | Lyn | 102 | 2.54 |
| 97 | Fyb | 84.8 | 2.64 |
| 98 | Grn | 2150 | 2.28 |
| 99 | Htr2b | 34.4 | 2.14 |
| 100 | Arpc1b | 189 | 2.44 |
| 101 | Lilrb4 | 120 | 3.66 |
| 102 | Cd180 | 96.5 | 3.52 |
| 103 | C1qb | 1050 | 3.51 |
| 104 | Abcc3 | 135 | 3.43 |
| 105 | C1qa | 1380 | 3.42 |
| 106 | Plau | 271 | 3.25 |
| 107 | Stab1 | 517 | 3.12 |
| 108 | Colec12 | 305 | 3.11 |
| 109 | Capg | 98.8 | 2.93 |
| 110 | Plin4 | 184 | 2.87 |
| 111 | Ifi44 | 102 | 2.86 |
| 112 | B2m | 1460 | 2.83 |
| 113 | Blnk | 62.8 | 2.82 |
| 114 | Cyth4 | 133 | 2.82 |
| 115 | Lgals3 | 418 | 2.82 |
| 116 | Rnase4 | 292 | 2.82 |
| 117 | Lgals9 | 167 | 2.79 |
| 118 | Serping1 | 191 | 2.79 |
| 119 | Gp49a | 60.5 | 2.75 |
| 120 | Ncf1 | 161 | 2.75 |
| 121 | Pld4 | 137 | 2.7 |
| 122 | Ifi27l2a | 74.6 | 2.67 |
| 123 | Bst2 | 177 | 2.65 |
| 124 | Fam46c | 102 | 2.62 |
| 125 | Csf1r | 808 | 2.61 |
| 126 | Fcgr1 | 71.6 | 2.61 |
| 127 | Igsf6 | 28.7 | 2.61 |
| 128 | Dab2 | 109 | 2.56 |
| 129 | 4632428N05Rik | 223 | 2.53 |
| 130 | Psmb8 | 112 | 2.53 |
| 131 | Serpina3f | 23.6 | 2.53 |
| 132 | Ifi202b | 88 | 2.52 |
| 133 | Tifab | 34.4 | 2.52 |
| 134 | Anxa3 | 90.4 | 2.5 |
| 135 | Sepp1 | 844 | 2.47 |
| 136 | Lgals3bp | 406 | 2.46 |
| 137 | Spsb1 | 109 | 2.46 |
| 138 | Mt2 | 2540 | 2.45 |
| 139 | Ctla2b | 52.6 | 2.44 |
| 140 | Hmha1 | 146 | 2.41 |
| 141 | Ctss | 1140 | 2.4 |
| 142 | Olfml3 | 329 | 2.4 |
| 143 | Lcp2 | 33.1 | 2.39 |
| 144 | Ifi44l | 47.8 | 2.38 |
| 145 | Ifit1 | 78.3 | 2.38 |
| 146 | Serpine2 | 306 | 2.38 |
| 147 | Lgals1 | 618 | 2.37 |
| 148 | Pycard | 21.8 | 2.37 |
| 149 | Arhgdib | 110 | 2.36 |
| 150 | Tlr4 | 39.2 | 2.35 |
| 151 | Hpse | 146 | 2.34 |
| 152 | Hvcn1 | 92.2 | 2.34 |
| 153 | Mpeg1 | 2500 | 2.33 |
| 154 | Cyba | 208 | 2.32 |
| 155 | P2rx7 | 48.4 | 2.31 |
| 156 | Sla | 64.2 | 2.29 |
| 157 | Naip2 | 41.6 | 2.28 |
| 158 | Psmb9 | 83.8 | 2.28 |
| 159 | Irgm1 | 130 | 2.27 |
| 160 | Lyz2 | 2880 | 2.27 |
| 161 | H2-Q4 | 197 | 2.24 |
| 162 | Itpripl2 | 99.8 | 2.24 |
| 163 | Nupr1 | 122 | 2.24 |
| 164 | Oas1b | 26.4 | 2.23 |
| 165 | Sfpi1 | 49.8 | 2.23 |
| 166 | Chi3l1 | 506 | 2.22 |
| 167 | Lipa | 422 | 2.22 |
| 168 | Slfn2 | 37.8 | 2.22 |
| 169 | Tubb6 | 49.4 | 2.22 |
| 170 | Clec5a | 25.9 | 2.21 |
| 171 | Grap | 32.2 | 2.21 |
| 172 | Plcg2 | 61 | 2.21 |
| 173 | Ptplad2 | 42 | 2.21 |
| 174 | AI607873 | 44.5 | 2.2 |
| 175 | Litaf | 215 | 2.19 |
| 176 | Gna15 | 24.8 | 2.18 |
| 177 | Plscr2 | 64.6 | 2.18 |
| 178 | Serpine1 | 66.9 | 2.18 |
| 179 | Apbb1ip | 37.6 | 2.16 |
| 180 | Fabp5 | 154 | 2.16 |
| 181 | Hk3 | 61.5 | 2.16 |
| 182 | S100a6 | 236 | 2.16 |
| 183 | Serpina3m | 25.6 | 2.16 |
| 184 | Evi2a | 42.4 | 2.15 |
| 185 | Gbp3 | 155 | 2.15 |
| 186 | Rassf2 | 102 | 2.15 |
| 187 | Csf3r | 80.6 | 2.13 |
| 188 | H2-T23 | 161 | 2.13 |
| 189 | Adap2 | 59.5 | 2.12 |
| 190 | Fcgr4 | 30.7 | 2.12 |
| 191 | Kcnk6 | 34 | 2.12 |
| 192 | Slc7a7 | 58.8 | 2.12 |
| 193 | Clec2d | 107 | 2.11 |
| 194 | Cst7 | 22.2 | 2.11 |
| 195 | Fcrls | 28.6 | 2.11 |
| 196 | Apoe | 10200 | 2.1 |
| 197 | Trim30a | 71.8 | 2.1 |
| 198 | Slc11a1 | 56.1 | 2.08 |
| 199 | Adcy7 | 153 | 2.05 |
| 200 | Anxa4 | 145 | 2.05 |
| 201 | Gbp1 | 163 | 2.05 |
| 202 | Gbp2 | 150 | 2.05 |
| 203 | H2-T24 | 59.9 | 2.05 |
| 204 | Eif2ak2 | 166 | 2.04 |
| 205 | Tgfbr2 | 163 | 2.04 |
| 206 | Tnfrsf1a | 177 | 2.04 |
| 207 | Axl | 448 | 2.03 |
| 208 | Isg15 | 53.6 | 2.03 |
| 209 | Amz1 | 31.4 | 2.02 |
| 210 | 5430435G22Rik | 85.9 | 2.01 |
| 211 | Ifitm3 | 169 | 2.01 |
| 212 | Rsad2 | 58.7 | 2.01 |
| 213 | Cmklr1 | 30.9 | 2 |
| 214 | Ucp2 | 638 | 2.34 |
| 215 | Emp1 | 107 | 2.23 |
| 216 | Emilin2 | 23.6 | 2.06 |
| 217 | Parp14 | 163 | 2.02 |
