# Supplementary material for: Minocycline counter-regulates pro-inflammatory microglia responses in the retina and protects from degeneration
Source: J Neuroinflammation. 2015 Nov 17;12:209. doi: 10.1186/s12974-015-0431-4 (PMC4650866; doi:10.1186/s12974-015-0431-4)
Supplement: Additional file 3: Table S2. — Differentially expressed transcripts comparing light exposure versus light exposed plus minocyline treatment. (DOCX 80 kb) [file 12974_2015_431_MOESM3_ESM.docx]

## Supplementary Table 2 - Differentially expressed transcripts comparing light exposure versus light exposed plus minocyline treatment

| **Nr** | **Gene** | **Mean count** | **log2 FC** |
| --- | --- | --- | --- |
| 1 | Dsp | 176 | 3.66 |
| 2 | Krt12 | 1080 | 3.57 |
| 3 | Aldh3a1 | 467 | 3.33 |
| 4 | Krt5 | 651 | 3.32 |
| 5 | Tmprss11a | 45.1 | 3.08 |
| 6 | Dsc3 | 35.8 | 3.06 |
| 7 | Fbln1 | 182 | 3.04 |
| 8 | Calml3 | 131 | 2.99 |
| 9 | Myh11 | 695 | 2.93 |
| 10 | Lypd2 | 70 | 2.92 |
| 11 | Dsg1a | 36.2 | 2.9 |
| 12 | Aqp3 | 46.3 | 2.89 |
| 13 | Prb1 | 93.9 | 2.84 |
| 14 | Tyr | 305 | 2.8 |
| 15 | Dcn | 441 | 2.75 |
| 16 | Lypd3 | 27.2 | 2.72 |
| 17 | Edn3 | 213 | 2.71 |
| 18 | Sdc1 | 85.3 | 2.68 |
| 19 | Tacstd2 | 32.7 | 2.6 |
| 20 | Krt6a | 101 | 2.58 |
| 21 | Anxa8 | 30.2 | 2.54 |
| 22 | Slurp1 | 33.2 | 2.44 |
| 23 | Krt6b | 52.8 | 2.41 |
| 24 | Trim29 | 34.4 | 2.37 |
| 25 | Fam129a | 348 | 2.32 |
| 26 | 1600029D21Rik | 38.4 | 2.25 |
| 27 | Cldn4 | 20.6 | 2.25 |
| 28 | Gja1 | 733 | 2.24 |
| 29 | Tagln | 476 | 2.23 |
| 30 | Fam83a | 25.2 | 2.22 |
| 31 | Islr | 184 | 2.21 |
| 32 | Gjb2 | 18.8 | 2.19 |
| 33 | Prpmp5 | 73.2 | 2.16 |
| 34 | Muc4 | 71.6 | 2.12 |
| 35 | Mlana | 151 | 2.11 |
| 36 | Lum | 72.6 | 2.09 |
| 37 | Tspan10 | 149 | 2.04 |
| 38 | Bace2 | 143 | 2.01 |
| 39 | Arpc1b | 239 | -2.01 |
| 40 | Htr2b | 40.9 | -2.02 |
| 41 | Naip5 | 37 | -2.02 |
| 42 | Fyb | 107 | -2.03 |
| 43 | Grn | 2620 | -2.03 |
| 44 | Lyn | 129 | -2.04 |
| 45 | Crym | 1750 | -2.05 |
| 46 | Ly86 | 216 | -2.06 |
| 47 | Serpina3n | 697 | -2.06 |
| 48 | Cd33 | 92 | -2.07 |
| 49 | Rasal3 | 46.2 | -2.07 |
| 50 | Casp1 | 28.3 | -2.08 |
| 51 | Spp1 | 10200 | -2.08 |
| 52 | Xdh | 165 | -2.08 |
| 53 | P2ry6 | 42.8 | -2.09 |
| 54 | Ptprc | 133 | -2.09 |
| 55 | Anpep | 118 | -2.1 |
| 56 | Btk | 45.8 | -2.1 |
| 57 | Galnt6 | 45.5 | -2.1 |
| 58 | Havcr2 | 206 | -2.1 |
| 59 | Gpr34 | 266 | -2.11 |
| 60 | Pik3cg | 52.2 | -2.11 |
| 61 | Ctsz | 2150 | -2.12 |
| 62 | Pbk | 14.3 | -2.13 |
| 63 | Dock2 | 99.2 | -2.14 |
| 64 | Rac2 | 93 | -2.14 |
| 65 | Hpgds | 148 | -2.15 |
| 66 | Tnfaip8l2 | 42.3 | -2.15 |
| 67 | Fcgr1 | 86.8 | -2.17 |
| 68 | Hexb | 2890 | -2.17 |
| 69 | Irf5 | 75.1 | -2.17 |
| 70 | Ms4a7 | 80.2 | -2.17 |
| 71 | Rrm2 | 19.9 | -2.17 |
| 72 | Oasl2 | 235 | -2.18 |
| 73 | Fcgr3 | 250 | -2.19 |
| 74 | Itgam | 310 | -2.19 |
| 75 | Parvg | 101 | -2.19 |
| 76 | Pik3ap1 | 56.4 | -2.19 |
| 77 | Sash3 | 36.4 | -2.19 |
| 78 | Fcgr2b | 126 | -2.21 |
| 79 | Apoc1 | 85.4 | -2.22 |
| 80 | Tbxas1 | 47.6 | -2.22 |
| 81 | Vav1 | 98.5 | -2.22 |
| 82 | Bcl3 | 59.4 | -2.25 |
| 83 | Itgb2 | 205 | -2.25 |
| 84 | Syk | 96.4 | -2.26 |
| 85 | Tnfrsf1b | 175 | -2.26 |
| 86 | Ctsd | 34100 | -2.27 |
| 87 | Gusb | 668 | -2.28 |
| 88 | Unc93b1 | 297 | -2.28 |
| 89 | Arhgap30 | 117 | -2.29 |
| 90 | Gp49a | 73.6 | -2.29 |
| 91 | P2ry13 | 69.8 | -2.29 |
| 92 | Ms4a6b | 83.3 | -2.3 |
| 93 | Tlr7 | 192 | -2.32 |
| 94 | Cd53 | 462 | -2.35 |
| 95 | Irf8 | 135 | -2.35 |
| 96 | Nckap1l | 356 | -2.35 |
| 97 | AF251705 | 107 | -2.36 |
| 98 | Arhgap25 | 54.1 | -2.36 |
| 99 | Cdk1 | 35.6 | -2.37 |
| 100 | Cx3cr1 | 378 | -2.37 |
| 101 | Kif4 | 390 | -2.39 |
| 102 | Arl11 | 72.7 | -2.4 |
| 103 | Csf2rb | 88 | -2.4 |
| 104 | Plin2 | 567 | -2.41 |
| 105 | C1qc | 1180 | -2.43 |
| 106 | Glipr1 | 45.3 | -2.43 |
| 107 | Cebpa | 148 | -2.44 |
| 108 | Gm8221 | 36.4 | -2.44 |
| 109 | Klhl6 | 68.6 | -2.45 |
| 110 | Myo1f | 141 | -2.45 |
| 111 | Laptm5 | 618 | -2.48 |
| 112 | Wdfy4 | 62 | -2.49 |
| 113 | Csf2rb2 | 57.7 | -2.51 |
| 114 | 1300002K09Rik | 56.2 | -2.52 |
| 115 | Cxcr4 | 157 | -2.53 |
| 116 | Gatm | 702 | -2.53 |
| 117 | Trem2 | 487 | -2.54 |
| 118 | Cd22 | 71 | -2.56 |
| 119 | Nlrc5 | 90.4 | -2.56 |
| 120 | Wfdc17 | 50.1 | -2.56 |
| 121 | Fam46c | 121 | -2.58 |
| 122 | C3 | 274 | -2.6 |
| 123 | Cd300a | 84.2 | -2.63 |
| 124 | Fcer1g | 283 | -2.7 |
| 125 | Ly9 | 82.7 | -2.7 |
| 126 | Top2a | 66.3 | -2.72 |
| 127 | Cybb | 144 | -2.75 |
| 128 | Siglec1 | 158 | -2.78 |
| 129 | Apobec1 | 242 | -2.8 |
| 130 | Tyrobp | 913 | -2.81 |
| 131 | Tlr13 | 142 | -2.82 |
| 132 | Stab1 | 627 | -2.89 |
| 133 | Lcp1 | 319 | -2.9 |
| 134 | Abcc3 | 165 | -2.91 |
| 135 | Lcn2 | 519 | -2.92 |
| 136 | Emr1 | 512 | -2.97 |
| 137 | Mki67 | 126 | -2.97 |
| 138 | Cd84 | 203 | -2.99 |
| 139 | Mmp12 | 33.1 | -3 |
| 140 | Hal | 86.1 | -3.03 |
| 141 | Ctse | 81.4 | -3.04 |
| 142 | C3ar1 | 341 | -3.05 |
| 143 | Cd68 | 728 | -3.06 |
| 144 | Timp1 | 79.8 | -3.07 |
| 145 | Ms4a6c | 218 | -3.13 |
| 146 | Slc15a3 | 153 | -3.23 |
| 147 | Lilrb4 | 146 | -3.25 |
| 148 | Steap4 | 249 | -3.3 |
| 149 | Clec7a | 563 | -3.32 |
| 150 | Ms4a6d | 259 | -3.42 |
| 151 | Msr1 | 342 | -3.92 |
